# Implications of Oxidative Stress in Glioblastoma Multiforme Following Treatment with Purine Derivatives

**DOI:** 10.3390/antiox10060950

**Published:** 2021-06-12

**Authors:** Marta Orlicka-Płocka, Agnieszka Fedoruk-Wyszomirska, Dorota Gurda-Woźna, Paweł Pawelczak, Patrycja Krawczyk, Małgorzata Giel-Pietraszuk, Grzegorz Framski, Tomasz Ostrowski, Eliza Wyszko

**Affiliations:** 1 Institute of Bioorganic Chemistry, Polish Academy of Sciences, Noskowskiego 12/14, 61-704 Poznan, Poland; mplocka@ibch.poznan.pl (M.O.-P.); agaw@ibch.poznan.pl (A.F.-W.); d_gurda@ibch.poznan.pl (D.G.-W.); ppawelczak@ibch.poznan.pl (P.P.); giel@ibch.poznan.pl (M.G.-P.); framski@ibch.poznan.pl (G.F.); tostr@ibch.poznan.pl (T.O.); 2 MRC Laboratory of Molecular Biology, Francis Crick Avenue, Cambridge CB2 0QH, UK; krawczyk@mrc-lmb.cam.ac.uk

**Keywords:** purine derivatives, kinetin riboside, glioblastoma multiforme, oxidative therapy, ROS, oxidative imbalance, cell death, cancer cells, spheroids

## Abstract

Recently, small compound-based therapies have provided new insights into the treatment of glioblastoma multiforme (GBM) by inducing oxidative impairment. Kinetin riboside (KR) and newly designed derivatives (8-azaKR, 7-deazaKR) selectively affect the molecular pathways crucial for cell growth by interfering with the redox status of cancer cells. Thus, these compounds might serve as potential alternatives in the oxidative therapy of GBM. The increased basal levels of reactive oxygen species (ROS) in GBM support the survival of cancer cells and cause drug resistance. The simplest approach to induce cell death is to achieve the redox threshold and circumvent the antioxidant defense mechanisms. Consequently, cells become more sensitive to oxidative stress (OS) caused by exogenous agents. Here, we investigated the effect of KR and its derivatives on the redox status of T98G cells in 2D and 3D cell culture. The use of spheroids of T98G cells enabled the selection of one derivative—7-deazaKR—with comparable antitumor activity to KR. Both compounds induced ROS generation and genotoxic OS, resulting in lipid peroxidation and leading to apoptosis. Taken together, these results demonstrated that KR and 7-deazaKR modulate the cellular redox environment of T98G cells, and vulnerability of these cells is dependent on their antioxidant capacity.

## 1. Introduction

Glioblastoma multiforme (GBM) is the most widespread and major invasive brain tumor in adults [1]. Unfortunately, it remains beyond the reach of previous therapies, which gives patients a short therapeutic window [2]. Most patients with GBM die within 15 months of diagnosis [3], which is mostly caused by limited drug uptake in tumor cells and tumor resistance to chemotherapy [4]. Thus, there is an immense need to develop more effective targeted treatment that affects energy metabolism of GBM cells to enhance their responsiveness to drugs [1,5].

Current treatment options for glioblastoma are mainly a combination of surgical resection of the tumor, followed by radiotherapy and adjuvant chemotherapy [1]. GBM is known to show intratumor heterogeneity [6], and rapid tumor progression contributes to the molecular characteristics of this cancer. Thus far, three major signaling pathways have been identified as the most deregulated ones in glioblastoma, namely, the activation of the receptor tyrosine kinase (RTK)/Ras/phosphoinositide 3-kinase (PI3K) pathway and inhibition of the p53 and retinoblastoma protein (Rb) signaling pathways (The Cancer Genome Atlas Research Network, 2008 [1]). Identification of new agents potentially capable of exploiting the metabolic sensitivity of cancer cells and their effect on molecular markers of GBM is necessary and obligatory [5,6,7,8]. The efficacy of small compounds with a potential inhibitory effect on the key signaling pathways in cancer cells has been evaluated [5,9]. It is not clear whether the abovementioned abnormalities of molecular machinery of glioblastoma cells might result from the increased basal levels of reactive oxygen species (ROS) in these cells. However, this imbalance of oxygen delivery, capacity, and consumption induces a proinflammatory environment, cancer cell migration and proliferation, drug resistance, and escape from cell death [10,11]. Oxidative stress (OS) can damage and modify proteins, lipids, and DNA; however, glioblastoma cells can survive and adapt in such a hypoxic environment, which enables them to resist treatment [11].

Thus, there is a pressing need to design new therapeutic small-molecular-weight compounds that can modulate the redox status of GBM cells and induce cell death through oxidative stress and the apoptosis pathway.

Chloroquine, temozolomide, cannabidiol, berberine, and bromopyruvate are well-known anticancer drugs that affect the redox status of GBM cells. Most of these compounds are antimetabolites, which structurally resemble natural substrates and affect mitochondrial functioning through elevated ROS production [9]. Thus, they seem to be promising and demanded therapeutic tools. We focused our attention on small compounds that might have high potential due to their bioavailability and proapoptotic activity. There are findings that natural small compounds may influence various steps of intracellular signaling pathways that are crucial for cell growth, proliferation, and apoptosis. We found a connection between observed KR-induced rapid ATP depletion and the enzymatic reactions that KR is involved in, such as phosphorylation by adenosine kinase, a crucial enzyme of the purine salvage pathway [12]. The current paper demonstrates the impact of *N*^6^-furfuryladenosine (kinetin riboside; KR) and its newly designed derivatives, 8-azakinetin riboside (8-azaKR) and 7-deazakinetin riboside (7-deazaKR), on the redox status of T98G GBM cells. Our previous study confirmed the complexity of the mechanism of action of KR, and we determined its effect on mitochondrial bioenergetics in HepG2 cells [12]. KR exerts a powerful anticancer effect and has an impact on molecular pathways that are crucial for cell increase, proliferation, and induction of cell death [12,13]. It is also a member of the purine analogue family, in which every compound may show an inimitable mechanism of action in neoplastic cells.

By performing molecular docking, flow cytometry analysis, confocal microscopy visualization, and bioluminescent determination of the ATP content, we assessed particular cellular parameters such as inhibition of cell proliferation and apoptosis induction and showed the effect of KR and its derivatives on oxidative imbalance. We analyzed the induction of metabolic (generalized OS and selective ROS detection and determination of glutathione level) and genotoxic OS after treatment with the abovementioned compounds. The analyses were performed using T98G cell culture (two-dimensional, 2D) and T98G cell spheroids (three-dimensional, 3D), which offered an in vitro system that mimicked the cancer cell environment and exhibited significantly stronger effect of the compounds than those achieved in monolayer cultures. The use of T98G cell spheroids was the first step of this research study, which enabled the screening and selection of one derivative—7-deazaKR—with comparable anticancer activity to KR. Detailed analyses comparing the pro-oxidative properties of these two adenosine analogues were performed using monolayer cultures of T98G cells.

Taken together, these results demonstrate that KR and 7-deazaKR are effective anticancer agents and might be promising alternatives in oxidative therapy by focusing on the cellular redox environment of GBM cells and induction of apoptosis.

## 2. Materials and Methods

### 2.1. Materials

KR, menadione, and carbonyl cyanide 3-chlorophenylhydrazone (CCCP) were purchased from Merck (Darmstadt, Germany).

### 2.2. Homology Modeling of Semi-Open Human Adenosine Kinase Conformation

For molecular docking, a semi-open conformation of adenosine kinase (ADK) was used, as large substitutions at N^6^ of adenosine (such as the furfuryl group present in KR) are known to inhibit the complete closure of the binding site of ADK, and phosphorylation of such substrates is performed in a semi-open state [14,15]. The only available structure of ADK in a semi-open conformation is *Toxoplasma gondii* ADK complexed with *N*^6^-dimethyladenosine (PDB code: 2A9Y). To obtain a human model of ADK in a semi-open conformation, homology modeling was performed using *T. gondii* ADK as a template. Sequence of the human ADK (PDB code: 1BX4) was used as a sequence of the target protein. In both structures (2A9Y and 1BX4), all the crucial protein–ligand interactions in the catalytic site are conserved [14,15,16]. To prepare 2A9Y for modeling, ligands present in the structure were removed, and reconstruction of missing atoms was performed using the Swiss PDB Viewer (SPDBV) program [17]. Almost all water molecules were removed, except for four molecules (HOH6014, HOH6045, HOH6051, and HOH6054), which were at 3.5 Å distance from 5′OH of *N*^6^-dimethyladenosine in the structure of 2A9Y. Preserving crucial water molecules has been shown to improve docking simulations in general [18] and, here, it stabilized the position of ligand during molecular docking, thus preventing incorrect interactions between 5′OH of KR analogues and G315 of 2A9Y. The prepared structure of 2A9Y was then used for alignment with 1BX4 sequence, and homology modeling was performed, using Modeler 9.18 [19] for both steps. Before minimizing the obtained model, hydrogen atoms were added to protein residues, and the remaining water molecules and their positions were optimized with PROPKA3 [20] using the following conditions: no pKa calculation, Amber forcefield, ensuring that novel atoms are not rebuilt nearby the occurring atoms, optimization of the hydrogen bonding network, and assigning charges to the ligand specified in a MOL2 file (reference ligand: 26A from 2A9Y with hydrogen atoms previously added using Chimera [21]). Next, a complex of model and reference ligand was minimized to ensure better orientation of residues in the catalytic site toward the ligand. The antechamber module of AmberTools17 [22] was used for the reference ligand to assign atom types and calculate atomic charges with the AM1-BCC method [23]. Next, the missing parameters were determined with the parmchk module of AmberTools17. Topology and coordinate files for the reference ligand and its complex with protein were created using the leap module of AmberTools17. The general Amber force field [24] was used for the compound and the ff99SB force field [25] was used for the protein. During minimization, positional restraints of backbone atoms were applied with a restraint force constant of 500 kcal/mol·Å^−2^. Minimization was performed in an implicit solvent, and a pairwise generalized Born model [26,27] was used with the first 250 steps being the steepest descent followed by 750 steps of conjugate gradient. A cutoff of 16 Å was applied, and a periodic boundary with constant volume was used.

### 2.3. Ligand Preparation and Molecular Docking

The 3D structures of KR and adenosine analogues were originally accessed from the ZINC12 database [28] and later used to build their *N*^6^-furfuryl derivatives by using GaussView from Gaussian 03 suite [29]. Then, pdbqt files used during docking for the final model and ligands were created using AutoDockTools 1.5.6 [30,31]. AutoDock Vina was used for docking calculations [32]. The search space was set to 22.5 × 15 × 15 Å with the default spacing of 0.375 Å, and coordinates of the center were set at −9, −41, and −106 in the dimensions of *x*, *y*, and *z*, respectively. Figures were prepared with AutoDockTools [30,31].

### 2.4. Synthesis of 8-azaKR and 7-deazaKR

The KR derivatives were synthesized according to the protocol described by Baranowski et al. [33]. Chemical structures of the compounds were drawn using ACD/ChemSketch (2017) Freeware.

### 2.5. Cell Culture

Human GBM cells (T98G) acquired from ATCC (American Type Culture Collection, Manassas, VA, USA) were cultured in Eagle’s Minimum Essential Medium (EMEM) (Corning^®^, New York, NY, USA) complemented with 10% fetal bovine serum (FBS, Corning^®^, New York, NY, USA) and antibiotics (ATCC, Manassas, VA, USA) at 37 °C in 5% CO_2_ atmosphere. Cells grown in supplemented media without the addition of KR and its derivatives were used as a negative control for all experiments. To analyze energy metabolism, comparative oxidative stress measurements were used. HepG2 cells purchased from ECACC (European Collection of Authenticated Cell Cultures, Salisbury, UK) were grown in EMEM (Corning^®^, New York, NY, USA) supplemented with 10% FBS (Corning^®^, New York, NY, USA) and antibiotics (ATCC, Manassas, VA, USA) and cultured under the same conditions as the T98G cell line.

### 2.6. Oxygen Consumption Analysis

HepG2 and T98G cells were seeded on T75 cell culture flasks and cultured in EMEM supplemented medium at 37 °C, in the atmosphere of 5% CO_2_, until the cells reached 80% confluency. Next, the cells were washed once with PBS and harvested using a cell scraper. The collected cells were counted, centrifuged (1000 rpm, 3 min), and resuspended in EMEM supplemented medium. Subsequently, oxygen uptake of approximately 3–4 × 10^6^ cells was measured by the Clark-type oxygen electrode (Oxygraph+ system, Hansatech Instruments Ltd; Narborough Rd, Pentney, UK) in 1 mL of EMEM supplemented medium at 37 °C. After stabilization of the initial oxygen consumption rate, in order to induce resting state (state 4) and maximal respiration (state U) and exclude nonmitochondrial oxygen consumption, 1.5 μM oligomycin (Merck, Darmstadt, Germany), 3.5 μM carbonyl cyanide *p*-trifluoromethoxy phenylhydrazone (FCCP, Merck, Darmstadt, Germany), and 1.5 μM rotenone/antimycin A (Merck, Darmstadt, Germany) were added, respectively. The working concentrations of stressor compounds were verified experimentally and separately for each cell line. After recording, the cells were collected to estimate protein concentration. Briefly, the cells were centrifuged, resuspended in 50 μL of PBS, and lysed by three freeze–thaw cycles (freezing at −80 °C for 5 min, thawing at 37 °C for 5 min). The obtained lysates were centrifuged, cell remains were ejected, and the total protein concentration was evaluated spectrophotometrically at 280 nm. Respiration parameters were calculated as follows: basal respiration, state 4 and state U, as the difference between the initial, oligomycin-sensitive or FCCP-sensitive oxygen consumption rate and the nonmitochondrial oxygen consumption rate, phosphorylating state (state 3), as the difference between basal respiration and state 4, and spare respiratory capacity (SRC) as the difference between state U and basal respiration. The obtained results were normalized to 1 mg of protein.

### 2.7. Comparative Mitochondrial ROS Analysis by Flow Cytometry

HepG2 and T98G cells were seeded on 6-well plates at a density of 2.5 × 10^5^ cells per well and cultured in EMEM supplemented medium at 37 °C and 5% CO_2_ saturation for 24 h. The cells were then handled with the ROS inducer menadione (Sigma-Aldrich, St. Louis, MO, USA) at the final concentration of 10, 30, or 50 μM for 30 min. Next, the cells were trypsinized, rinsed twice with 1 mL of DPBS (Thermo Fisher Scientific, Waltham, MA, USA), and incubated with 1 μM MitoSOX Red probe (Invitrogen, Waltham, MA, USA) at 37 °C for 25 min in dark. Immediately after staining, the cells were analyzed with a FACSCalibur flow cytometer (Becton Dickinson, Franklin Lakes, NJ, USA).

### 2.8. Spheroid Formation, Treatment, and Labeling Preparation for Screening of KR Derivatives

T98G cells were seeded in 96-well U-bottom plates (Nunclon Sphera, Thermo Fisher Scientific, Waltham, MA, USA) at a density of 2 × 10^3^ cells per well in 200 µL EMEM (Corning^®^, New York, NY, USA). The plates were briefly centrifuged at 250× *g* (according to the manufacturer’s instructions) and then incubated at 37 °C under 5% CO_2_ conditions. After 72 h of maturation, the cell spheroids were treated with 80 and 200 µM KR, 8-azaKR, and 7-deazaKR for 24, 48, and 72 h. The spheroids were refed every 24 h by removing 100 µL of medium from each well and replacing it with 100 µL of fresh medium containing the appropriate concentration of the compound. At the end of each incubation time (24, 48, and 72 h), an analysis of spheroid viability and oxidative stress induction was performed. The production of ROS was observed to identify various parameters of OS contributing to the formation of generalized (cytoplasmic) OS and selective ROS (superoxide).

For spheroid imaging, the cells were rinsed with PBS, transferred to a fresh medium containing an appropriate fluorescent probe for labeling the target molecules, and incubated for appropriate time under growth conditions:

#### 2.8.1. LIVE/DEAD Analysis of T98G Spheroids

The LIVE/DEAD™ assay differentiates live cells from dead cells by simultaneous 15-min staining with green fluorescent calcein-AM (2 µM of final concentration) and red fluorescent ethidium homodimer-1 (4 µM of final concentration) to mark loss of plasma membrane integrity. Next, the spheroids were washed two times with PBS to remove any residual dye and then transferred to glass-bottom dishes for confocal microscopy and placed in FluoroBrite™ DMEM (Thermo Fisher Scientific, Waltham, MA, USA). Live cell images in Z-stack were collected by Leica TCS SP5 II confocal laser scanning microscope equipped with a white-light laser (470–670 nm) and an environmental cell culture chamber that provided controlled conditions of temperature, CO_2_ saturation, and humidity. Images were sequentially scanned and collected at 490/505–550 nm (green fluorescence) and 530/600–660 (red florescence) nm (±20) by using an HC PL APO 20×/0.75 water/oil-immersion objective with 1.5× digital zoom. Leica LAS AF 2.7.3 software was used to control image processing. For the fluorescence intensity analysis, Leica LAS X 3.3.3 software with a 3D deconvolution module was used. A *Z*-projection was created from Z-stacks using the “max” intensity option, and the ROI was then selected to measure the fluorescence intensity of spheroids. The results are presented as a mean of fluorescence intensity.

#### 2.8.2. Intracellular and Mitochondrial Oxidative Stress Measurements in T98G Spheroids

To detect the metabolic OS, the cell spheroids were rinsed with PBS, transferred to a fresh medium containing 5 µM H_2_DCFDA and 5 µM MitoSOX, and stained for 30 min. Additionally, for total ROS analysis, cell nuclei were labeled with 5 µg/mL Hoechst 33342 (Thermo Fisher Scientific, Waltham, MA, USA) for 5 min. Next, the spheroids were washed two times with PBS to remove any residual dye, transferred to glass-bottom dishes for confocal microscopy analysis, and placed in FluoroBrite DMEM (Thermo Fisher Scientific, Waltham, MA, USA). Live cell images in Z-stack were collected using a Leica TCS SP5 II confocal laser scanning microscope with a white-light laser (470–670 nm), an HC PL APO 20×/0.75 water/oil-immersion objective, and an environmental cell culture chamber. Sequentially scanned images were acquired at 498/505–550 nm (ex/em) for H_2_DCFDA, 514/570–630 nm (ex/em) for superoxide determination, and 405/430–480 nm (ex/em) for nuclear staining. Leica LAS AF 2.7.3 software was used to control image processing. For the fluorescence intensity analysis, Leica LAS X 3.3.3 software with a 3D deconvolution module was used. A *Z*-projection was created from Z-stacks using the “max” intensity option, and the ROI was then selected to measure the fluorescence intensity of the spheroids. The results are presented as a mean of fluorescence intensity.

### 2.9. Comparative Analysis of the Anticancer and Pro-Oxidative Properties of KR and 7-deazaKR by Using Two-Dimensional Culture of T98G Cells

#### 2.9.1. In Vitro Phosphorylation

Novel nucleoside analogues were evaluated as a substrate for adenosine kinase (ADK) in an in vitro phosphorylation assay (Precice ADK Assay Kit, NovoCIB, Lyon, France). The ADK-catalyzed phosphorylation reaction of 7-deazaKR and 8-azaKR was run for 30 min at 37 °C in a reaction buffer (100 mM Tris-HCl pH 8.5, 250 mM KCl, 10 mM MgCl2, 2.5 mM NAD, 2.75 mM ATP, IMPDH 20 mU/mL, and human recombinant ADK 2.2 mU/mL). The positive control of reaction efficiency was a phosphorylation reaction with 4 mM KR as a substrate, whereas, to inhibit reaction (negative control), 5-iodotubercidin (ADK inhibitor) was added to a final concentration of 500 µM. Phosphorylated products were separated and identified on fluorescent TLC Silica Gel 60 F254 plates (20 × 20 cm, Merck Millipore, Darmstadt, Germany) by using the following separation phase: ammonia/isopropanol/water (2:7:1 *v*/*v*). After resolution, the plates were dried, and the products were detected using the Gel Doc-it Imaging System (UVP, Upland, CA, USA). The phosphorylated products were quantified using Multi Gauge V3.0 (Fujifilm, Tokyo, Japan) software for Windows.

#### 2.9.2. Bioluminescent Measurement of Cellular ATP Content

T98G cells were seeded at the density of 3 × 10^5^ cells/well onto 6-well plates, cultured in growth medium (EMEM) at 37 °C and 5% CO_2_ saturation, and incubated until 70–80% confluency was reached. Then, the cells were treated with KR and 7-deazaKR at the final concentration of 80 and 200 µM for 6 h. Next, the cells were separated by trypsin, washed twice with 1 mL of DPBS, and centrifuged, and the pelleted cells were resuspended in 100 µL PBS. Freeze–thaw cycles (−80 °C for 5 min and then at 37 °C) were used to prepare cell lysates. This step was repeated three times. After centrifugation, cell debris was extruded, and the total protein content was measured spectrophotometrically at 280 nm. Quantitative determination of ATP (with recombinant firefly luciferase and its substrate d-luciferin) was performed using the Molecular Probes^®^ ATP Determination Kit (Thermo Fisher Scientific, Waltham, MA, USA) according to the manufacturer’s protocol. Luminescence was measured at 560 nm using the VICTOR Nivo multimode plate reader (Perkin Elmer, Walthman, MA, USA).

#### 2.9.3. Real-Time Analysis of Cell Proliferation Using the xCELLigence System

Cell proliferation analysis was performed using the xCELLigence system (Roche, Basel, Switzerland). In brief, 100 µL medium was added to E-plates for measuring offset values, and then T98G cells were seeded in an extra 50 µL of medium at a density of 4 × 10^3^ cells per well. The cells were allowed to attach to the E-plates at 37 °C and 5% CO_2_ saturation in a cell incubator for 30 min before the insertion into the xCELLigence platform. After 24 h, the cells were treated with KR and 7-deazaKR at the final concentration of 80 and 200 µM in an additional 50 µL of medium. Simultaneously, T98G cells were treated with a combination of ribosides and 1 µM 5-iodotubercidin (an ADK inhibitor). Control cells were cultured in the supplemented medium without desired compounds. The real-time monitoring of the proliferation of treated T98G cells was monitored at 30 min intervals from the time of plating for 96 h. The real-time proliferation of the cells was estimated by impedance measurement and expressed as a cell index (CI) value; normalization values were registered using RTCA Software 1.2.1.

#### 2.9.4. Apoptosis/Necrosis Assay by Flow Cytometry

The apoptosis/necrosis assay was performed in T98G cells (monolayer culture) by staining with CellEvent™ caspase 3/7–FITC (Thermo Fisher Scientific, Waltham, MA, USA) and propidium iodide (PI, Merck, Darmstadt, Germany) fluorescent dyes with excitation/emission at 503/530 nm and 535/617 nm, respectively. The cells (3 × 10^5^) were seeded onto 6-well plates containing the growth medium (EMEM), cultured at 37 °C and 5% CO_2_ saturation, and incubated until 70–80% cell confluency was achieved. After a day, the cells were treated for 24 h with KR and 7-deazaKR at the final concentration of 40, 80, and 200 µM. Subsequently, T98G cells were detached with trypsin and washed twice with 1 mL of DPBS (1 mL) (Thermo Fisher Scientific, Waltham, MA, USA). Subsequently, the cells were harvested and suspended in a solution containing CellEvent™ caspase 3/7–FITC (10 µM) and PI (3 µg/mL) in accordance with the manufacturer′s protocol for 30 min at 37 °C in dark. The cells were analyzed promptly after staining with excitation at 488 nm by the FACSCalibur flow cytometer (Becton Dickinson, Franklin Lakes, NJ, USA).

#### 2.9.5. Metabolic OS (Intracellular and Mitochondrial) Measurement by Flow Cytometry

T98G cells were seeded (3 × 10^5^ cells/well) onto 6-well plates and cultured in EMEM at 37 °C and 5% CO_2_ saturation until 70–80% cell confluency was reached. Subsequently, the cells were exposed for 24 h to the tested compounds at the final concentration of 40, 80, and 200 µM. The intracellular ROS level was analyzed by staining with H_2_DCFDA Reagent (ex/em: ~492–495/517–527 nm) in accordance with the manufacturer’s protocol (Thermo Fisher Scientific, Waltham, MA, USA), wherein ROS trigger the fluorescence. The cells were separated by trypsin and washed twice with DPBS (1 mL). The pelleted cells were suspended in 0.5 mL of DPBS containing H_2_DCFDA at the final concentration of 0.5 µM. The cells were then incubated at 37 °C for 30 min in dark. The abovementioned cell culture environment was also created for measuring the mitochondrial OS (superoxide level) induction by flow cytometric analysis. After treatment with KR and its analogue, the cells were detached with trypsin and washed with DPBS (1 mL). Cell pellets were stained with MitoSOX (2.5 µM) for 10 min at 37 °C in dark. The cells were analyzed after incubation with the dyes, with excitation at 488 nm by the FACSCalibur flow cytometer (Becton Dickinson, Franklin Lakes, NJ, USA).

#### 2.9.6. Lipid Peroxidation Measurements by Flow Cytometry

T98G cells were seeded at a density of 3 × 10^5^ cells/well onto 6-well plates and cultured in the same environmental conditions as the above experiments. Then, the cells were treated for 24 h with the tested compounds (40, 80, and 200 µM). Next, the cells were separated with trypsin and washed twice with DPBS (1 mL), and intracellular oxidation of lipids was analyzed by staining with BODIPY^®^ 581/591 C11 reagent according to the manufacturer’s protocol (Thermo Fisher Scientific, Waltham, MA, USA). Upon oxidation in living cells, the reagent shifts the fluorescence emission peak from 590 nm (red) to 510 nm (green). The cells were analyzed promptly after incubation at 488 nm excitation by using the FACSCalibur flow cytometer (Becton Dickinson, Franklin Lakes, NJ, USA), and data were analyzed by FlowJo software. The ratios of the signal from the 590 to 510 channels were used to determine lipid peroxidation in cells.

#### 2.9.7. Glutathione Level Measurement by Flow Cytometry

T98G cells were seeded at a density of 3 × 10^5^ cells/well onto 6-well plates and in the same environmental conditions as the above experiments. Next, the cells were treated with KR and 7-deazaKR at the final concentration of 40, 80, and 200 µM for 24 h. The cellular glutathione (GSH) level was measured by staining with the nonfluorescent Thiolite Green dye, according to the manufacturer’s protocol (AAT Bioquest, Sunnyvale, CA, USA) that emits strong fluorescence after reaction with thiols (ex/em: ~540/590 nm). After incubation with the compounds, the cells were detached with trypsin and washed twice as described above. Subsequently, the cells were suspended in a staining solution and incubated at 37 °C in 5% CO_2_ environment for 30 min. After staining, the fluorescence intensity was analyzed using the FACSCalibur flow cytometer with excitation at 488 nm.

#### 2.9.8. Analysis of 8-oxo-dG in T98G Cells by HPLC-UV-ED

T98G cells (1 × 10^6^ cells/mL) were seeded onto T25 flasks and cultured in standard environmental conditions for 24 h. Subsequently, the cells were exposed to 80 and 200 µM KR and 7-deazaKR for 24 h. After incubation, the cells were detached and washed twice with PBS. Total DNA was isolated from the treated cells and untreated control cells by using the Genomic mini DNA isolation kit (A&A Biotechnology, Gdańsk, Poland) according to the manufacturer′s protocol. Quality of the total DNA was assessed spectrophotometrically.

Analysis of 8-oxo-dG in T98G cells by HPLC-UV-ED was performed as described by Barciszewska et al. [34]. The total amount of 8-oxo-dG in the genome was calculated using a special formula described in [34].

#### 2.9.9. Total RNA Isolation

T98G cells were seeded at the density of 1.5 × 10^5^ cells/well onto 12-well plates and cultured in EMEM at 37 °C and 5% CO_2_ saturation until 70–80% cell confluency was achieved. Subsequently, the cells were treated for 24 h with the tested compounds at the final concentration of 40, 80, and 200 µM. Total RNA from the treated T98G cells was isolated using the TRIzol^®^ Reagent (Invitrogen, Waltham, MA, USA) according to the manufacturer’s protocol. DNA residue was removed with DNase I (DNA-free DNA Removal Kit, Thermo Fisher Scientific, Waltham, MA, USA). The total RNA concentration was measured using a NanoDrop 2000 UV/Vis spectrophotometer at 260 nm.

#### 2.9.10. cDNA Synthesis and Real-Time

Total RNA (0.5 µg) was used for cDNA synthesis with the Transcriptor First-Strand cDNA Synthesis Kit (Roche, Basel, Switzerland) using oligo (dT) primers following the manufacturer’s description. Real-time PCR analysis was performed to determine the expression levels of the SOD, CAT, GSS, SESN1, SESN2, NRF2, NFKB, SIRT2, PGC1, PARP, TFA, and p53 genes. Each cDNA sample was analyzed using Mono Color Hydrolysis UPL Probes (Roche, Basel, Switzerland) selected for each gene by using ProbeFinder Software (Roche, Basil, Switzerland). The PCR reaction mixtures were prepared in line with the manufacturer’s protocol. PCR conditions for all genes were as follows: initial incubation step at 94 °C for 10 min, followed by 45 cycles of amplification (15 s at 94 °C, 30 s at 60 °C, and 15 s at 72 °C) (single acquisition), with a final cooling step at 40°C for 2 min. The analysis was performed using a LightCycler 480 II instrument (Roche, Basel, Switzerland). Relative gene expression was calculated using the Roche Applied Science E-Method and normalized to the reference genes ACT, TBP, PGK1, and HPRT1. All standard curves were generated by amplifying a series of twofold dilutions of cDNA. The primer sequences for the analyzed genes and UPL probes are shown in Table 1.

### 2.10. Statistical Analysis

Statistical analysis was accomplished using GraphPad Prism version 8.0 for Windows (GraphPad Software, San Diego, CA, USA). Two-way ANOVA by Tukey’s multiple comparison test was used for the fluorescence intensity analysis of spheroids after MitoSOX, LIVE/DEAD, and H_2_DCFDA staining. To determine the significance of flow cytometric analyses of cellular OS, glutathione level, 8-oxo-dG content, and lipid peroxidation, one-way ANOVA followed by Dunnett’s multiple comparison test was used; for superoxide level measurement, one-way ANOVA followed by Bonferroni’s correction was used. The significance of ATP depletion was determined by one-way ANOVA followed by Tukey’s comparison. The importance of cell viability by apoptosis/necrosis assay was determined by two-way ANOVA followed by Tukey’s multiple comparison test, and oxygen consumption significance was evaluated by Student’s *t*-test. One-way ANOVA followed by Dunnett’s multiple comparison test was used for the gene expression level analysis. The results are presented as mean ± SD obtained from three independent biological replicates with three experimental repeats for each. A *p*-value < 0.05 was considered statistically significant.

## 3. Results

### 3.1. Small Compounds as a Part of Oxidative Therapy in GBM Cells: Metabolic Profiles and Comparative Analysis of Mitochondrial ROS in T98G vs. HepG2 Cells

**Figure 1:**
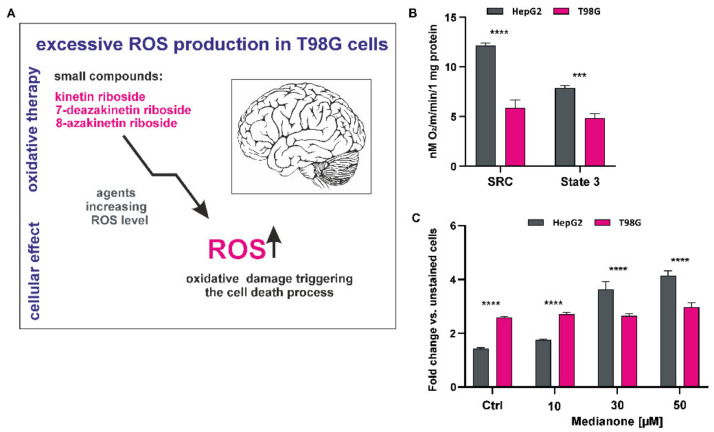
Aerobic status of glioblastoma (T98G) and non-glioblastoma (HepG2) cells. (**A**) Involvement of small compounds in the oxidative therapy of T98G cells. (**B**) Comparison of spare respiration capacity (SRC) and phosphorylating state (state 3) of T98G cells (gray) vs. HepG2 cells (magenta) by using the Oxygraph+ system. (**C**) Flow cytometry analysis of comparative mitochondrial oxidative stress induction in T98G cells (gray) and HepG2 cells (magenta) after menadione treatment. Fluorescence intensity shift is presented as a bar graph (mean ± SD) of three independent experiments. Statistical significance is indicated with asterisks: *** *p* < 0.001, **** *p* < 0.0001.

Cellular redox status is a balance of oxygen delivery, capacity, and consumption, and it prevents cell oxidative damage. ROS are produced constantly during cellular respiration and stimulate different signaling pathways in cancer cells [35]. GBM cells are known to have high metabolic rate and produce a high level of ROS, which promotes tumor progression and drug resistance. The easiest method to induce cell death in GBM cells is to reach the redox threshold and circumvent the antioxidant defense mechanisms. Consequently, cells become more sensitive to OS caused by external agents (Figure 1A). In our study, we tested three naturally occurring analogues of adenosine that enhance ROS levels in a T98G cell line. We assumed that these compounds might be promising options in oxidative therapy that focused on the cellular redox environment of GBM and induction of apoptosis (Figure 1). First, we determined the energetic status of HepG2 and T98G cells by respiration measurements using a Clark-type oxygen electrode. The respiration rate of HepG2 cells was significantly higher than that of T98G cells, exceeding 50% in basal respiration and reaching almost 80% in maximal respiration. Our results revealed that HepG2 cells had 62.5% more intense oxidative phosphorylation (OXPHOS) and 106.8% greater reserve in mitochondrial capacity than T98G cells (Figure 1B). These findings suggest higher glycolytic activity in T98G cells, including anaerobic ATP production level.

Mitochondria are one of the main sources of ROS, which are mainly generated in the form of superoxide anions (O_2_^−^) as a byproduct of oxidative metabolism and contribute to mitochondrial damage [36]. Comparative mitochondrial OS induction by menadione, a commonly used ROS inducer, in T98G and HepG2 cells, was investigated by flow cytometry using MitoSOX dye. The fluorescence of the dye (red) is triggered selectively in the mitochondria in the presence of superoxide (Figure 1C). Analysis after 30 min of treatment with increasing concentrations of menadione revealed that the analyzed compound induced superoxide production in HepG2 cells and affected them significantly with almost threefold higher level of superoxide production. The T98G cells showed increased basal levels of ROS without any changes in fluorescence shift after menadione induction; this might be related to drug resistance and activation of the antioxidant defense mechanism by cancer cells (Figure 1C).

### 3.2. Determination of KR Analogues Showing Similar Affinity for ADK

**Figure 2:**
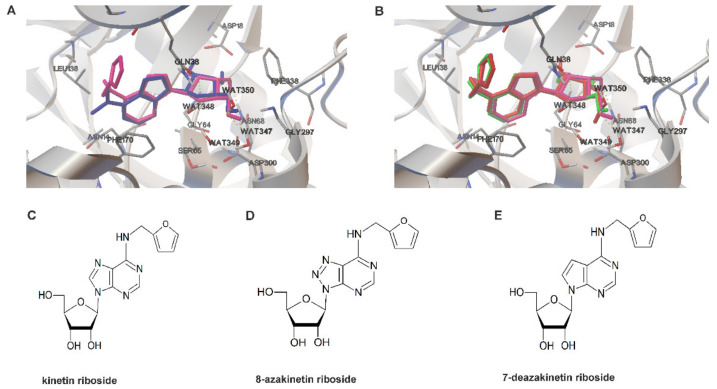
Determination of kinetin riboside analogues featuring similar affinity for adenosine kinase. (**A**) Juxtaposition of docked poses of KR (magenta) and the reference ligand dimethyladenosine (blue) in the binding cavity of the modeled semi-open conformation of human ADK structure represented by ribbons. Hydrogen bonds are depicted as yellow dotted lines. Residues in close contact to ligands are shown as sticks. (**B**) Juxtaposition of docked poses of KR (magenta), 8-azaKR (red), and 7-deazaKR (green) in the binding cavity of the modeled semi-open conformation of human ADK structure represented by ribbons. Hydrogen bonds are depicted as yellow dotted lines. Residues in close contact to ligands are shown as sticks. (**C**–**E**) Structure of kinetin riboside and its two derivatives, 8-azakinetin riboside and 7-deazakinetin riboside, with similar affinity binding to human ADK.

The estimated binding energy calculated by AutoDock Vina for the reference ligand dimethyladenosine from 2A9Y was −9.4 vs. −9.2 for KR. Both conformers were generally well superimposed with all the crucial protein–ligand interactions being preserved (Figure 2A). Therefore, it can be assumed that the presence of the *N*^6^-furfuryl group in KR did not impair the ability of the molecule to adopt a proper conformation and be accommodated in the binding cavity. Moreover, the docking results suggest that the *N*^6^-furfuryl group of KR can form an additional hydrophobic interaction with the side-chain of Leu138. Two compounds, namely, 8-azakinetin riboside (8-azaKR) and 7-deaza kinetin riboside (7-deaza-KR), were predicted to dock particularly well in the binding cavity of the ADK model (Figure 2B). Moreover, the extra nitrogen atom in 8-azaKR was predicted to form additional polar interactions with side-chains of Cys123 and Gln38. The estimated binding energies calculated by AutoDock Vina for both derivatives were lower than that for KR, with values of −9.6 and −9.3 for 8-azaKR and 7-deazaKR, respectively, indicating that the two KR derivatives might have similar affinity to ADK as KR alone. Interestingly, 8-azaadenosine and some adenosine analogues containing a 7-deaza ring (sangivamycin and toyocamycin) are known to be phosphorylated by ADK and further incorporated into DNA and RNA, which contributes to their anticancer properties [37,38,39]. This characteristic makes the two adenine ring modifications particularly attractive to test in the context of KR derivatives. Furthermore, it has been shown that 8-azaadenosine is rapidly deaminated inside the cells and that cytotoxicity of 8-azaadenosine is enhanced when cells are pretreated with an adenosine deaminase inhibitor [37]. The presence of the furfuryl group at N^6^ of 8-azaKR would most likely prevent the deamination reaction, which could contribute to increased potency of 8-azaKR inside the cells in comparison to 8-azaadenosine. Derivatives of KR were obtained according to the previously reported procedure for the synthesis [33] (Figure 2C–E).

### 3.3. Impact of KR, 8-azaKR, and 7-deazaKR Treatment on the Viability of T98G Spheroids

**Figure 3:**
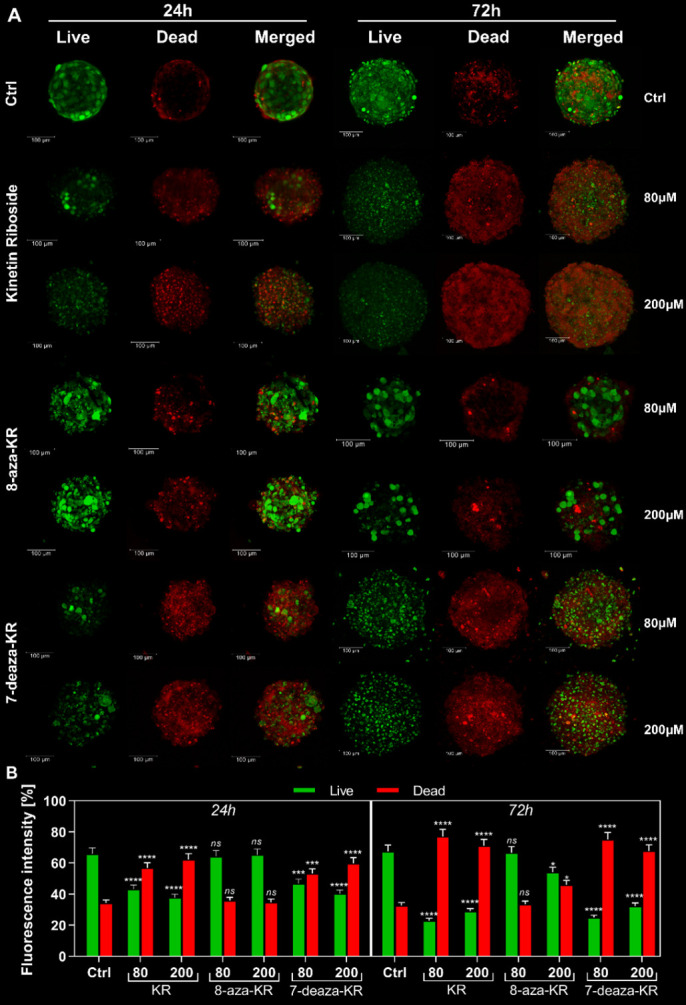
Viability analysis of T98G spheroids after treatment with KR and 7-deazaKR treatment. (**A**) Confocal microscopy analysis of the viability of T98G cells in 3D culture by using the LIVE/DEAD assay kit. Green (ex/em: 490/505–550 nm) and red fluorescence (ex/em: 530/600–660 nm) correspond to live and dead cells, respectively. Merged images are shown on the right panels. (**B**) Analysis of the fluorescence intensity of spheroids. The results are presented as mean ± SD of three independent measurements. Statistical significance (two-way ANOVA): (ns) not significant, * *p* < 0.05, *** *p* < 0.001, **** *p* < 0.0001.

The T98G cell spheroid culture model that mimics the extracellular microenvironment of the tumor was used to obtain more precise prediction of the in vivo results of compound rating and was the first step to screen KR derivatives. The viability of cells within the spheroids was analyzed using the LIVE/DEAD^®^ Viability/Cytotoxicity Kit by confocal microscopy (Figure 3A), and fluorescence intensity was then estimated (Figure 3B). Staining with ethidium homodimer-1 was used to indicate a loss of cell membrane integrity (red fluorescence), while calcein AM fluorescence demonstrated metabolically viable cells (green fluorescence). We investigated the distribution of living and dead cells after treatment with 80 and 200 μM KR, 8-azaKR, and 7-deazaKR; the results revealed that all three compounds affected cell viability in a time- and dose-dependent manner. After 24 h incubation, the most potent effect was visible after KR and 7-deazaKR treatment, regardless of the concentration, which was observed as an increase in red fluorescence from dead cells (Figure 3A). Moreover, inhibition of cell proliferation and rupture of the outer layer of cell spheroids were observed. The observed minimal changes (not statistically significant) in the red fluorescence intensity of the dye showed that 8-azaKR did not interfere with the viability of the treated spheroids. The percentage of dead cells (ethidium homodimer-1-stained) after KR and 7-deazaKR treatment increased with more than a twofold change in fluorescence intensity as compared to that for untreated cells for the highest doses of the compounds (Figure 3B). The cell viability analysis after 72 h revealed that KR and 7-deazaKR at 80 μM concentration showed a strong effect (Figure 3A). Moreover, after treatment with 200 μM concentration of these compounds, the structure of the spheroid was already relaxed and less compact, which might lead to spheroid breakdown and leakage of the dyes. Treatment with 200 μM 8-azaKR for 72 h caused a slight increase in the fluorescence intensity as compared to that for untreated cells which remained viable; however, cell death occurred (Figure 3A,B).

### 3.4. Induction of the Intracellular and Mitochondrial OS in T98G Spheroids after Treatment with KR, 8-azaKR, and 7-deazaKR

**Figure 4:**
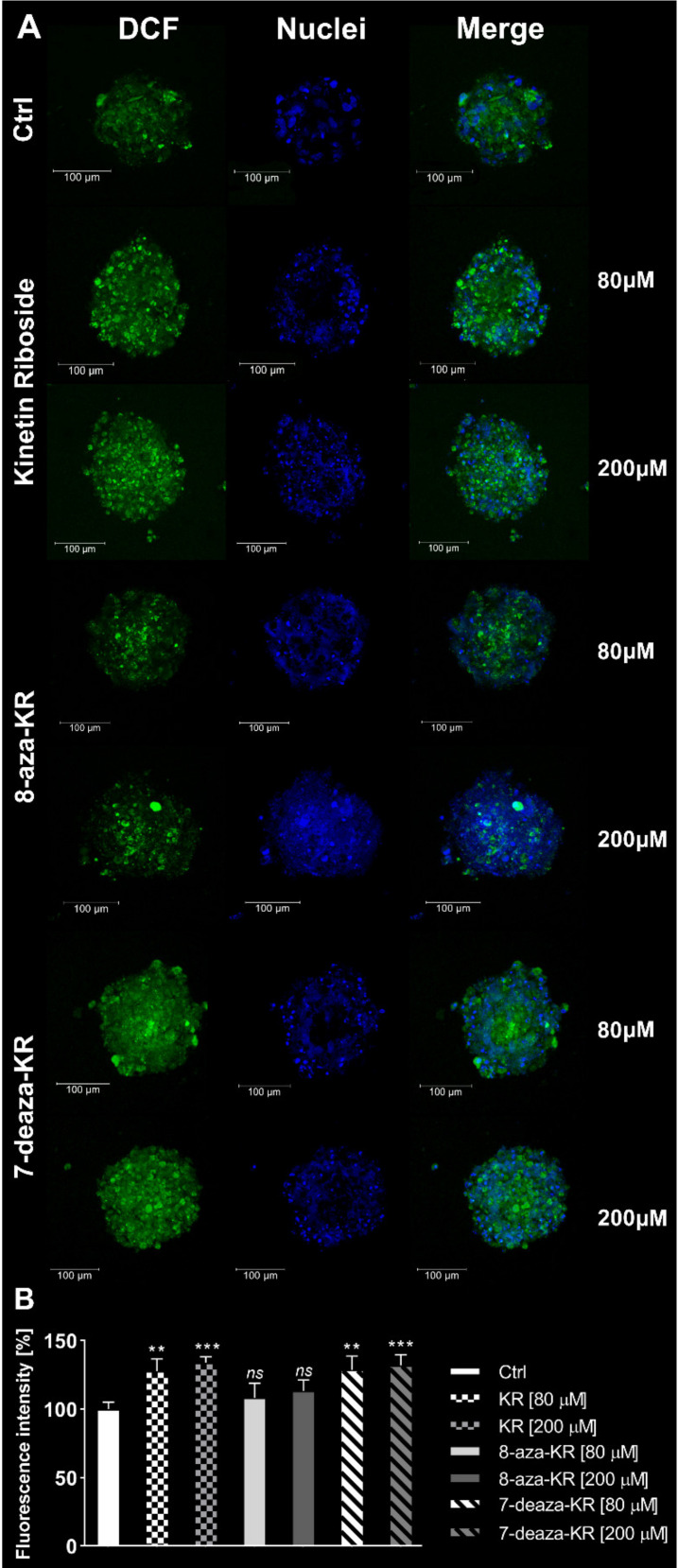
Cellular oxidative stress analysis in T98G 3D cell cultures. (**A**) Confocal microscopy analysis was performed after 24 h of treatment with 80 and 200 µM of KR and 7-deazaKR. Oxidative stress was determined by H_2_DCFDA staining (ex/em: 498/505–550 nm). Nuclei were stained with Hoechst 33342 (ex/em: 405/430–480 nm). Connected images are presented on the right panel. (**B**) Analysis of the shift in the fluorescence intensity of spheroids after treatment. The results are presented as the mean fluorescence intensity ± SD of three measurements. Statistical significance (two-way ANOVA): (ns) not significant, ** *p* < 0.01, *** *p* < 0.001.

To determine and confirm whether treatment with 80 and 200 μM KR, 8-azaKR, and 7-deazaKR induces cytoplasmic ROS generation, we performed confocal microscopy analysis of the T98G cell spheroids double stained with 2′,7′-dichlorodihydrofluorescein diacetate (H_2_DCFDA) and Hoechst 33342 for cell nuclei (Figure 4). H_2_DCFDA forms a fluorescent compound after reaction with ROS. The 24 h treatment presented an increase in fluorescence derived from the oxidized dye up to approximately 30% at the highest concentrations of KR and 7-deazaKR, thus showing the induction of OS (Figure 4A,B). We observed minimal changes (not statistically significant) in the fluorescence intensity of the dye after 8-azaKR treatment, which was comparable to that for the untreated spheroids, thus indicating that 8-azaKR did not interfere with the redox status of the treated spheroids.

**Figure 5:**
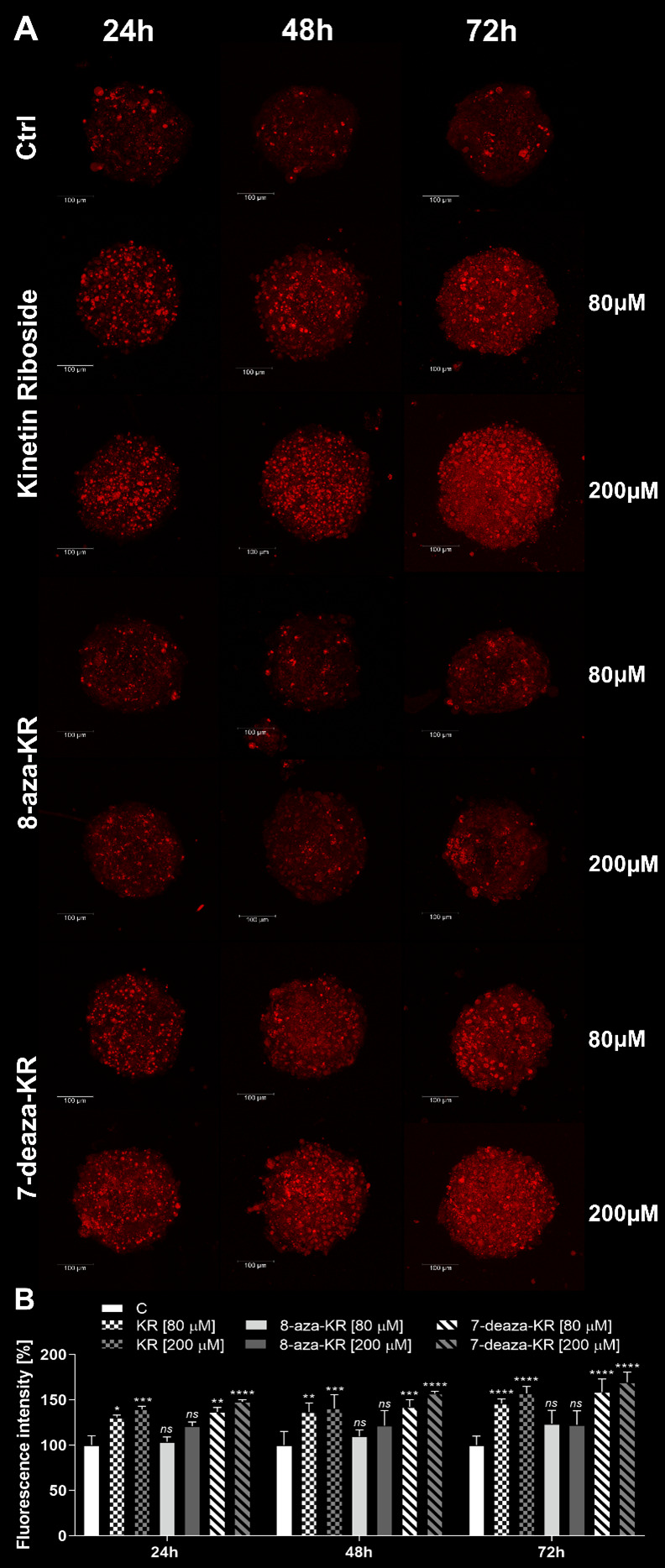
Mitochondrial oxidative stress analysis in T98G spheroids. (**A**) Confocal microscopy analysis of the mitochondrial oxidative stress after 24, 48, and 72 h of treatment with KR, 8-azaKR, and 7-deazaKR (80 and 200 µM) determined by MitoSOX staining (ex/em: 510/570–600 nm). (**B**) Analysis of changes in the fluorescence intensity of spheroids. The results are presented as the mean fluorescence intensity ± SD of three independent experiments. Statistical significance (two-way ANOVA): (ns) not significant, * *p* < 0.05, ** *p* < 0.01, *** *p* < 0.001, **** *p* < 0.0001.

Subsequently, we analyzed the mitochondrial OS induction by all three compounds in T98G spheroids by confocal microscopy. The presence of superoxide in the mitochondria is reflected by emission of red fluorescence by MitoSOX dye. Our analysis showed that KR, 8-azaKR, and 7-deazaKR affected mitochondrial redox homeostasis in a dose- and time-dependent manner (Figure 5). We observed that treatment with 80 and 200 µM KR and 7-deazaKR efficiently induced superoxide production and that the effect was more potent and apparent with increasing incubation time (Figure 5A). After 72 h of treatment with both compounds, we observed an increase in fluorescence emitted by the oxidized dye up to approximately 50% as compared to that for untreated cells (Figure 5B). The incubation of cells for 24 and 48 h with 8-azaKR did not show a spectacular increase in mitochondrial OS, regardless of the concentration. The effect was eventually observed after 72 h of treatment and slightly increased as compared to that for control cells (Figure 5A,B).

Taken together, the use of T98G spheroids allowed us to select one KR derivative with anticancer activity similar to that of KR, and it also showed the comprehensive effects of these compounds. We selected 7-dezaKR for further and detailed analyses to compare its effect with that of KR and to explore the potential of both these compounds in the oxidative therapy of GBM. 

### 3.5. ADK Is Required for Complete Activity and Toxicity of the Adenosine Derivative through the Salvage Pathway. Treatment with KR and 7-deazaKR Induces Rapid Depletion of Cellular ATP Levels, Leading to T98G Cell Death

**Figure 6:**
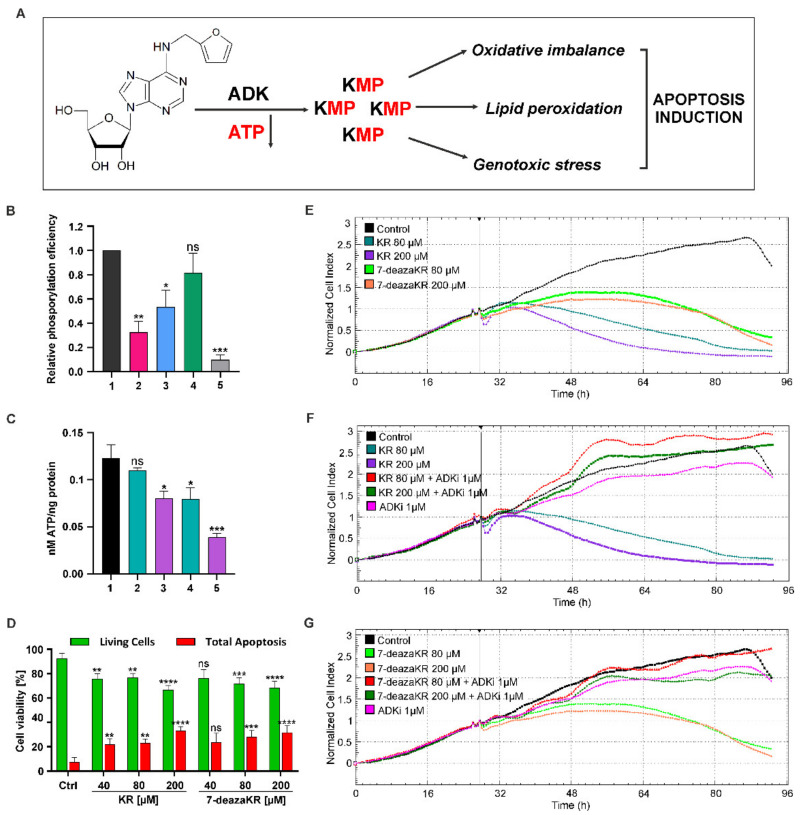
Kinetin riboside and 7-deazakinetin riboside activity and toxicity in T98G cells depend on ADK activity. (**A**) Phosphorylation of kinetin riboside by ADK promotes its cellular toxicity, leading to apoptosis induction. (**B**) In vitro phosphorylation of 7-azaKR (1–4 mM, bars 2–4, respectively) by ADK. The data are presented as the mean ± SD of three independent experiments. Bar 1—positive control of phosphorylation efficiency with 2 mM KR as a substrate. For the negative control, 0.5 mM 5-iodotubercidin was used as an ADK inhibitor (bar 5). (**C**) Determination of ATP level in the cells after treatment with 80 and 200 µM KR and 7-deazaKR treatment (bars 2–5, respectively) compared to control (bar 1). The results normalized for 1 mg of protein are shown as the mean ± SD from three independent experiments. (**D**) Flow cytometry analysis of apoptosis/necrosis in T98G cells after 24 h incubation with KR and 7-deazaKR (40–200 µM). (**E**–**G**) T98G real-time cell proliferation in the presence of KR and 7-deazaKR. The influence of KR and 7-deazaRK (80 and 200 µM) on HepG2 cell proliferation (**E**) with the addition of an ADK inhibitor (1 µM iodotubercidin; (**F**,**G**) was monitored by the xCELLigence system for 120 h at 30 min intervals. The results are representative of at least three independent experiments. Green bars indicate live cells, while red bars represent cells with both early and late apoptosis. The data are presented as the mean percentage ± SD from three independent experiments. Statistical significance is indicated with asterisks: (ns) *p* > 0.05, * *p* < 0.05, ** *p* < 0.01, *** *p* < 0.001, **** *p* < 0.0001.

Our goal was to demonstrate that KR and 7-deazaKR are natural anticancer agents and might be promising alternatives, with a focus on the cellular redox environment of glioblastoma and induction of apoptosis in abnormal cells through the activation of the salvage pathway of purine biosynthesis (Figure 6A). We started from the in vitro phosphorylation (reaction) of the following ribosides by ADK (Figure 6B) and performed thin-layer chromatography to observe the effect of this process. For KR, we used a previously determined and optimal concentration of 2 mM (data not shown), while, for its new derivative, we performed a concentration selection test during this reaction. We then established the relative values of this reaction in relation to the formed metabolite—kinetin riboside monophosphate (KMP). The analysis showed that the most efficient phosphorylation occurs when the highest concentration (4 mM) of 7-deazaKR is used (the phosphorylation of the derivative was 18.5% lower than that for KR), but the effect was proportionally stronger with increasing concentrations. Moreover, the use of an adenosine kinase inhibitor, 5′-iodotubercidin, reversed the reaction. The inhibitor concentration was also determined earlier, and the most optimal concentration was chosen (Figure 6B). In order to estimate the cellular ATP content, we performed a bioluminescence assay which revealed the decrease in cellular ATP level in T98G cells treated with both compounds (Figure 6C). An instant drop in ATP level was detected after 6 h, and the ATP level depleted by more than 33% following exposure to 200 μM KR; the effect was more rapid with a significant decrease in ATP level for treatment with 200 μM 7-deazaKR, where the depletion of ATP level reached 75%. Interestingly, treatment with 80 μM 7-deazaKR led to a 42% drop in ATP level, which is comparable to that achieved with the highest concentration of KR (Figure 6C). The optimal concentrations of the compounds and time conditions followed by ATP determination were selected, and extension of the incubation time would not result in a pronounced drop in ATP. Subsequently, we investigated real-time cell proliferation using the xCELLigence system and estimated cell apoptosis by flow cytometry (Figure 6E–G). We used the xCELLigence instrument for tracking T98G cell growth to compare the toxic effect of KR and 7-deaza KR. Viability of T98G cells was monitored for 96 h every 30 min (Figure 6E), and treatment with the compounds was performed after 24 h of cell growth. The kinetics of cell viability measurement supplied transient information about the influence of the tested compounds. We observed a meaningful decrease in the CI value of T98G cells that occurred after 24 h treatment with 200 μM KR, while its derivative only slowed the proliferation of cells and showed a less spectacular effect. The derivative exhibited a weaker effect on the inhibition of cell proliferation when compared with the lower concentration of KR (Figure 6E). This indicated that T98G cells were sensitive to both compounds, but the index and velocity of the reaction were different. Moreover, to demonstrate once again the importance of ADK phosphorylation of ribosides, we used its inhibitor (1 μM 5′-iodotubercidin) and proved that this is a crucial step for obtaining toxic effects of the compounds. The inhibitor itself (magenta line on the graph) is not toxic to cells and efficiently stopped phosphorylation, which did not lead to a decrease in the cell proliferation rate (Figure 6F,G). 

The two main and well-established pathways leading to cell death are apoptosis and necrosis. To examine whether KR and its derivative induced apoptosis in T98G cells, we carried out flow cytometric analysis by using CellEvent^®^ caspase-3/7 Green ReadyProbes^®^ Reagent and PI dual staining. KR and 7-deazaKR influenced T98G cells’ viability and proliferation in a dose- and time-dependent manner (Figure 6D). A 24 h exposure to 40–200 μM KR elevated the percentage of apoptotic cells (caspase-3/7/PI) to 25.8% in comparison to the control cells (7.34 %), whereas, after treatment with 7-deaza KR, the effect was similar to that for KR, and it was 24.4% for the highest doses of the compounds (Figure 6D).

Taken together, these results imply that rapid and marked ATP depletion is one of the early consequences of exposure to purine derivatives and leads to loss of cell viability.

### 3.6. Disruption of the Oxidative Parameters in T98G Cells after KR and 7-deazaKR Treatment: The Effect of the Compounds on the Activation of Metabolic OS and Antioxidant Defense Mechanism

**Figure 7:**
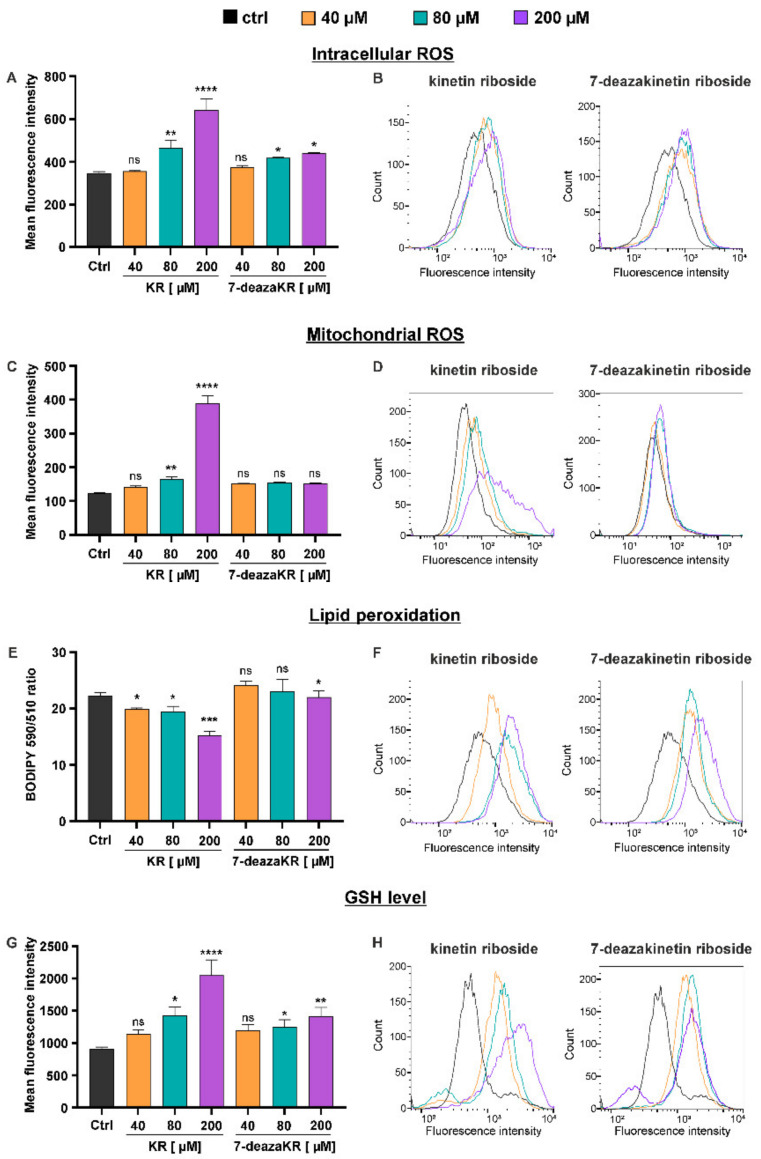
Influence of kinetin riboside and 7-deazakinetin riboside on oxidative stress parameters in T98G cells. (**A**,**B**) Intracellular ROS detection in the cells after KR and 7-deazaKR treatment (40–200 µM). ROS production was examined by flow cytometry using H_2_DCFDA staining, and the fluorescence intensity was estimated. Data are shown as a bar graph of three independent experiments (mean ± SD, A) or as representative histograms (**B**). (**C**,**D**) Flow cytometry analysis of mitochondrial OS induction in T98G cells after KR and 7-deazaKR treatment (40–200 µM) by using the MitoSOX fluorescent indicator. Fluorescence intensity shift was plotted in a bar graph (mean ± SD; C) and is presented as a representative histogram of three independent experiments (**D**). (**E**,**F**) Induction of lipid peroxidation in T98G cells after KR and 7-deazaKR treatment (40–200 µM) analyzed by flow cytometry. Upon oxidation, the emission fluorescence of the BODIPY 581/591 probe shifts from 590 to 510 nm; the 590/510 ratio of fluorescence intensity is presented as a bar graph (mean ± SD) from three individual experiments (**E**). Fluorescence shift is also shown as representative histograms (**F**). (**G**,**H**) A simultaneous analysis of cellular GSH content measured by staining with the nonfluorescent Thiolite™ Green dye. The fluorescence intensity changes are given in a bar graph of three independent experiments (mean ± SD; (**G**) or presented as representative histograms (**H**). Statistical significance is indicated with asterisks: (ns) *p* > 0.05, * *p* < 0.05, ** *p* < 0.01, *** *p* < 0.001, **** *p* < 0.0001.

In the next step, we analyzed whether metabolic ROS at the pathological level disrupts the cells causing the damage of proteins, lipids, and nucleic acids. Cytoplasmic ROS level was evaluated by H_2_DCFDA staining through the flow cytometric assay (Figure 7A,B). T98G cells treated with the increasing concentration of both compounds for 24 h exhibited an increase in fluorescence emitted by the dye up to 85% at the highest concentration of KR, whereas it was approximately 30% for treatment with 200 μM 7-deazaKR (Figure 7A). The increase in the fluorescence intensity of the dye reflects the induction of OS, as observed in flow cytometry histograms (Figure 7B). We also observed that KR and 7-deazaKR treatment entailed the activation of the mitochondrial OS in T98G cells (MitoSOX staining) by flow cytometry (Figure 7C,D). Our results indicate that 200 µM KR sharply enhanced mitochondrial superoxide generation, whereas the impact was not so significant at lower concentrations (Figure 7C). Furthermore, regardless of concentration, 7-deazaKR caused cells to show an increase in the fluorescence to more than approximately 1.3-fold as compared to control cells (Figure 7C). The generation of the mitochondrial ROS in T98G cells is also shown on the flow cytometry histograms as a fluorescence shift (Figure 7D).

Intense OS is usually accompanied with the degradation of lipids and, ultimately, may cause direct damage of the cell membrane [40,41]; therefore, we evaluated the induction of lipid peroxidation after treatment with KR and 7-deazaKR (Figure 7E,F). The rate of lipid peroxidation was estimated using the reagent 581/591 C11 that localizes in the membrane of living cells. We estimated that the 590/510 ratio, based on red and green florescence data obtained by flow cytometry, was inversely proportional to the amount of peroxided lipids (Figure 7E). The analysis revealed that only KR actively caused lipid peroxidation and the decrease in the estimated ratio was proportional to the increasing concentration of the compound. KR at 200 µM concentration showed the highest peroxidation rate, as indicated by the lowest 590/510 ratio (decrease of approximately 32% compared to the untreated T98G cells; Figure 7E). 7-deazaKR-treated cells exhibited lipid peroxidation comparable to that of the untreated cells, and the compound caused a slight decrease in the 590/510 ratio only at the highest concentration. The activation of lipid peroxidation in T98G cells is also shown on the flow cytometry histograms as a single wavelength of the red florescence shift (Figure 7F). Increase in the red florescence intensity is correlated with the amount of oxidized lipids, which is in contrast with the ratio analysis.

We also observed that treatment with KR and 7-deazaKR forced the natural antioxidant defense systems of T98G cells to protect against excessively formed ROS (Figure 7G,H). An increase was observed in the reduced GSH content, which was measured by staining with the nonfluorescent Thiolite Green dye. The increase in cellular GSH concentration after treatment with 200 μM KR was the most significant, and this was correlative with the increase in fluorescence to more than 2.3-fold as compared to that in control cells (Figure 7G). The effect of the KR derivative was similar and comparable to that of 80 μM KR, where we observed a 1.6-fold change in the increase in fluorescence intensity (Figure 7H).

These results firmly suggest that OS elicited by KR and 7-deazaKR can be the trigger of T98G cell apoptosis (Figure 7). Furthermore, KR showed a greater effect on the mitochondrial OS induction with simultaneous activation of lipid peroxidation.

### 3.7. Effect of KR and 7-deazaKR on DNA Oxidation

**Figure 8:**
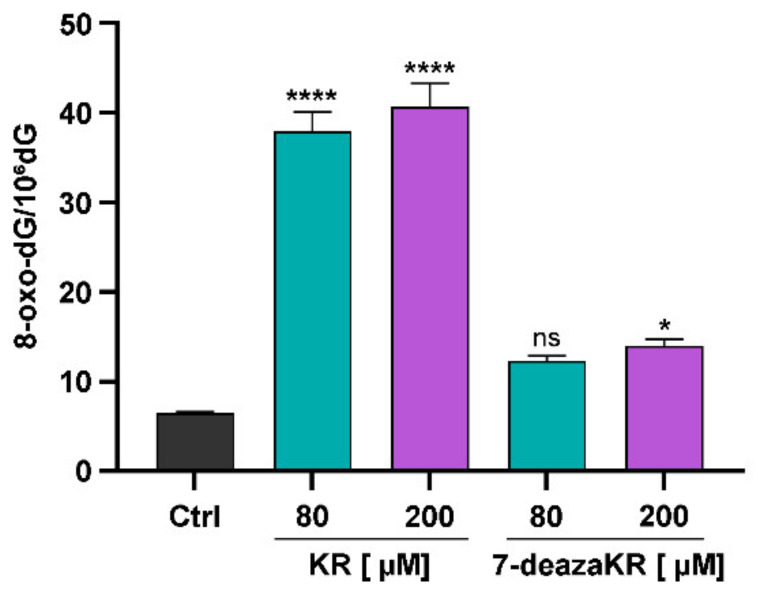
Quantitative analysis of 8-oxo-dG content in T98G cells by HPLC-UV-ED after treatment with KR and 7-deazaKR. The number of 8-oxo-dG residues per 1 × 10^6^ base pairs in DNA was calculated in cells after 24 h incubation with KR and 7-deazaKR (80 and 200 µM). Control cells were cultured in fully supplemented growth medium alone. Statistical significance is indicated with asterisks: (ns) *p* > 0.05, * *p* < 0.05, **** *p* < 0.0001.

We additionally evaluated the level of intracellular ROS by determining the content of 2′-deoxy-8-oxoguanosine (8-oxo-dG) in the enzymatic DNA hydrolysates obtained from T98G cells incubated with the tested compounds. 2′-Deoxyguanosine is considered to be the most susceptible compound to oxidation among the four canonical nucleosides, and 8-oxo-dG is the major oxidation product in DNA. The content of 8-oxo-dG was measured by HPLC-UV-ED, and the number of 8-oxo-dG molecules per 10^6^ dG was calculated as shown in the diagram (Figure 8A). Treatment with KR significantly elevated the number of 8-oxo-dG molecules as compared to that for untreated control, and treatment at both concentrations (80 and 200 μM) exhibited more than 5.5- to 5.7-fold higher numbers of 8-oxo-dG molecules in T98G cells (37.89 ± 2.205 and 40.725 ± 2.58 per 10^6^ dG, respectively). 7-deazaKR showed a mild effect on the number of 8-oxo-dG molecules; for 80 and 200 μM concentration, it was respectively 12.325 ± 0.575 and 14.035 ± 0.710 per 10^6^ dG as compared to that for untreated cells.

### 3.8. Effect of KR and 7-deazaKR on the Expression Level of Genes Involved in Oxidative Stress Response in T98G Cells

**Figure 9:**
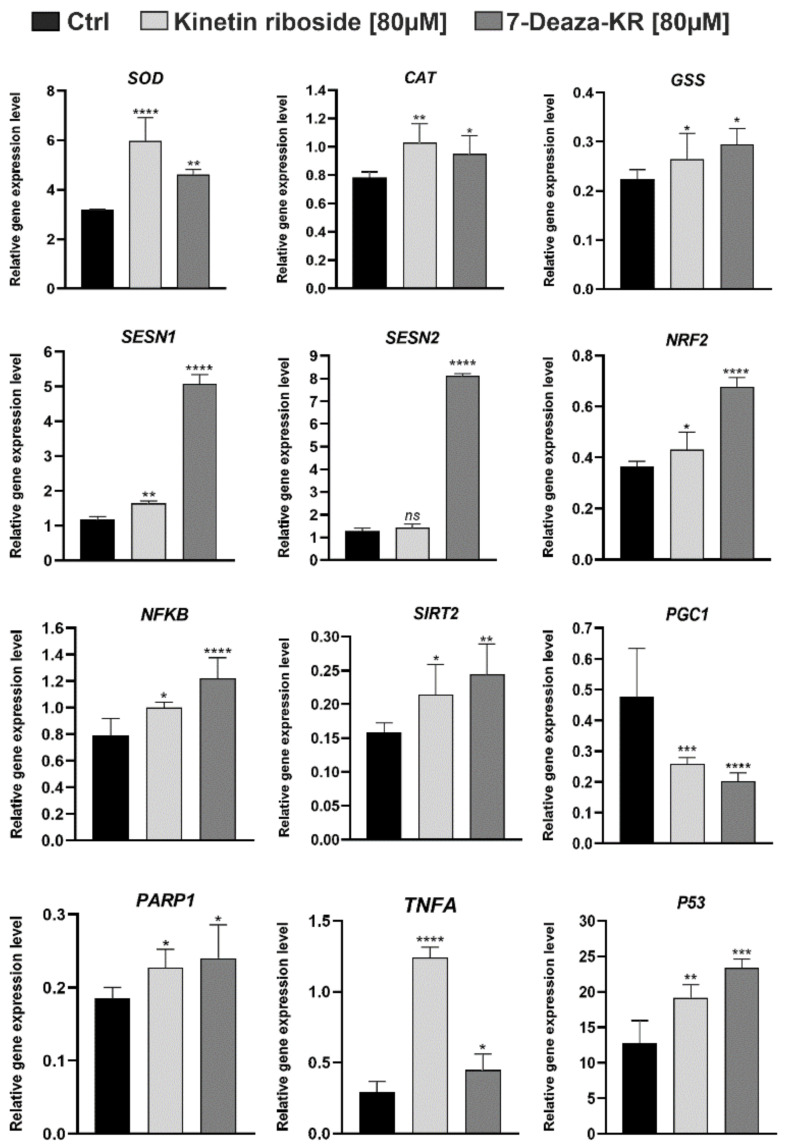
The expression level of selected genes analyzed in 2D cell culture of T98G cells treated with KR and 7-deaza-KR at the final concentration of 80 µM. Relative real-time PCR analysis was performed for the genes SOD, CAT, GSS, SESN1, SESN2, NRF2, NFKB, SIRT2, PGC1, PARP, TFA, and p53 24 h after treatment. The results are presented as the mean ± SD obtained from three biological replicates and three independent experimental repeats for each one. Statistical significance (one-way ANOVA): (ns) not significant, * *p* < 0.05, ** *p* < 0.01, *** *p* < 0.001, **** *p* < 0.0001.

Lastly, we analyzed the changes in the gene expression levels of several enzymes related to oxidative stress and cellular welfare indicators in the presence of KR and 7-deazaKR (Table 2; Figure 9). We found that both KR and 7-deazaKR activated enzymatic scavengers involved in the antioxidant defense (SOD, CAT, and GSS) at similar levels. After treatment with both compounds, we observed an increase in the expression levels, which might be related to the protection against excessively formed ROS. The most significant change was observed for superoxide dismutase (SOD), which was correlated with an increase in the expression to approximately more than threefold change as compared to that for control cells (Figure 9). However, we noted that 7-deazaKR had a greater effect on genes related to oxidative stress, such as SESN1, SESN2, NRF2, and NFKB, and it significantly increased the expression level of these genes as compared to that noted for KR. The expression level of SESN2 remained unchanged after treatment with KR. We also observed that both derivatives decreased the expression level of critical regulator of mitochondrial energy metabolism, PGC-1a, which in turn caused the activation of other genes belonging to the group of the cellular welfare indicators (SIRT2, PARP1, TNFA, and p53) (Figure 9). KR showed a greater influence on the expression level of *TNFA*, which was more than a fivefold change as compared to that for control cells (Figure 9).

## 4. Discussion

GBM is the primary and most invasive nervous system tumor in the adult population [1], with a characteristic genetic heterogeneity [42]. Glioblastomas can be classified as either primary or secondary [6]; however, different subtypes exist, and the most used classification method of these tumors is a grading scheme [41]. The most frequently occurring tumors of GBM are primary tumors, which develop from normal glial cells; however, there is an evidence that neural stem cells (NSCs) and oligodendrocyte precursor cells (OPCs) might also be precursors of GBM [43]. It has also been shown that low-grade gliomas tend to differentiate, but only grade 4 gliomas lead to glioblastoma and gliosarcoma, which represent the rifest forms with a high expression of malice. These tumors are considered to be the most aggressive ones because of the great level of microvascular proliferation and necrosis, with a high tendency to metastasize to the brain [41].

Unfortunately, despite significant efforts and a multitude of investigations, including studies based on RNAi technology [44,45], GBM remains beyond the reach of effective therapies, which leads to poor prognosis of patients and a high rate of mortality [2]. Intensive cellular proliferation, abnormal formation of vascular structures [41], and deregulation of crucial signaling pathways in cancer cells [1] might be some of the major factors responsible for the resistance of cancer cells to standard treatments [7]. Recently, an immense need has emerged for individualized therapy development; thus, many targeted drugs have been investigated. Moreover, studies on the identification of molecular markers of GBM have also been conducted. The first attempts were made to evaluate the efficacy of small compounds as potential inhibitors of key signaling pathways in GBM cells [5]. To date, temozolomide (TMZ) is considered as a promising pharmaceutical candidate in the context of targeted therapy of patients with glioblastoma, and it is frequently combined with other small inhibitors. The combined treatment with TMZ and curcumin showed an inhibitory effect on autophagy in GBM cells through the activation of the NF-κB and PI3K/Akt pathways [46]. Furthermore, the combination of TMZ with resveratrol affected the activation of AMPK and inhibited mTOR (the mechanistic target of rapamycin), leading to apoptosis of glioblastoma cells [47]. The list of agents targeting different growth factor pathways that are frequently activated in glioblastoma cells is still expanding, with the addition of new ones that are more potent and specific to glioblastoma [6].

Cancer cells develop various mechanisms to escape cell death and induce a high proliferation rate through their ability to repair DNA damage, cell-cycle arrest, variations in the expression of oncogenes, induction of autophagy, hypoxia [48], and alterations in tumor metabolism [12]. Glioblastoma cells have an increased level of ROS, which results from the impairment of the mechanisms related to the production and elimination of ROS [40]; these impairments are mainly based on the mitochondrial dysfunction or inefficient antioxidant systems [42]. ROS are produced in all types of cells, and there are several constitutive sources responsible for its production, among which mitochondria are the primary ones [49]. Furthermore, changes in redox homeostasis that contribute to cancer development and progression might also derive from the transition of metal ions, peroxisome activity, endoplasmic reticulum stress, or oxidase activity [41,49]. The “oxygen economy” imbalance in GBM is closely linked to the environmental factors that promote tumor growth, differentiation, and survival. OS is a triggering factor of various pathological processes, including modification of cellular components and crucial biomolecules, consequently leading to genotoxicity [40,41]. Thus, OS promotes the induction of hypoxia and forces cells to adapt to such conditions, which results in the resistance of cancer cells to treatment [11]. It has been shown that stem cells in the tumor mass might be related to the resistance of GBM because of their reinforced protection against OS [42].

Following the development of hypoxia in the tumor microenvironment, metabolic activities of cancer cells are altered comparative to normal cells [41]. These modifications support the maintenance of malignant properties and create an intrinsic cell resistance mechanism associated with “metabolic reprogramming” [12]. Regardless of the elevated ROS level, GBM cells can survive in such environments by relying on lactic acid fermentation, which is associated with the high rate of anaerobic glycolysis [49]. This metabolic strategy can provide adequate intermediates for the biosynthesis of nucleotides and amino acids [50], and it is also involved in ROS detoxification through a decrease in OXPHOS activity [12,49].

To confirm these observations, we performed a comparative analysis of energy metabolism in human hepatocellular carcinoma cells (HepG2) and a human glioblastoma cell line (T98G) (Figure 1B,C). The HepG2 cell line possesses appropriate characteristics for in vitro experiments involving energy imbalance and induction of oxidative stress [12], and the T98G cell line is routinely utilized as an experimental standard for improving therapeutic strategies for GBM [51]. A high-resolution, comparative analysis of oxygen consumption by intact cells revealed that HepG2 cells rely on mitochondrial respiration, and that their utilization of oxygen is instant and more rapid than that by T98G cells (Figure 1B). The analysis showed that state 3 (oligomycin-inhabitable respiration), that is, the consumption of oxygen associated with ATP synthesis, is higher in HepG2 cells; this indicates a greater potential of these cells to enter the OXPHOS pathway (Figure 1B). We showed a high level of SRC in HepG2 cells, which might constitute a greater adaptation of these cells to mitochondrial metabolism. In contrast, a low level of SRC in T98G cells causes consistent depletion in glioblastoma cells, which possibly indicates OS and a high level of proliferation rate [52] (Figure 1B). This confirms that the metabolism of T98G cells mainly relies on anaerobic glycolysis, which leads to the transformation of glioblastoma cells and maintains the balance between the production and protection against ROS in a nontoxic range. This ability based on the glucose-induced inhibition of cell respiration is termed as the Crabtree effect and supports the survival of cancer cells, leading to drug resistance and affecting the testing of chemotherapeutic agents [53,54]. Our previous study also confirmed that glioblastoma cell lines undergo the Crabtree effect; we also showed this effect in an A172 cell line, which is another commonly used model for manifesting glioblastoma properties [12]. The metabolic flexibility of cancer cells may indicate tumor aggressiveness, and, to overcome this limitation, the presence of a mitochondrial oxidative phenotype is desirable, similar to that in HepG2 cells [52,55]. Moreover, gliomas possess mitochondrial structural abnormalities, genomic mutations in mtDNA, and altered energy metabolism; thus, some of the small compounds may indirectly modulate metabolic disturbances that are a consequence of mitochondrial dysfunction [56].

We also conducted a comparative analysis of mitochondrial ROS in HepG2 and T98G cells, with simultaneous supplementation of the OS factor menadione. The analysis revealed that T98G cells showed increased basal levels of ROS, which did not change after menadione treatment, whereas HepG2 cells responded to the treatment and OS was induced (Figure 1C). This might indicate that the redox state of GBM cells protects them from apoptosis, thus creating a favorable environment for cellular proliferation and inducing drug resistance [10,57].

The commonly identified genetic aberrations in glioblastoma include overexpressed and often mutated p53, PTEN (phosphatase and tensin homolog deleted on chromosome ten), VEGF (vascular endothelial growth factor), EGFR (epidermal growth factor receptor), and the PI3CA pathway. Unfortunately, the use of inhibitors of these targets in a personalized therapeutic approach still has limitations in terms of treatment and clinical success [1]. Thus, many other promising targeted therapies have been recently developed, including small compounds that might modulate the redox status of glioblastoma cells [10]. Most of these compounds act through the activation of the intracellular ROS production in cancer cells, which finally leads to cell death mediated by OS and activation of the apoptotic and necrotic pathways [58]. GBM cells possess a high basal metabolic rate [41]; therefore, disrupting the cellular redox status and stability (generation vs. scavenging of ROS) may sensitize the GBM cells to drugs (Figure 1A) [40]. The group of anticancer drugs that affect the redox balance of the GBM cells and destroy malignant cells is expanding, and there are already a few well-established drugs such as chloroquine [59], temozolomide [60], cannabidiol [41], berberine [61], and bromopyruvate [62].

Natural small compounds are also considered to selectively influence various stages of intracellular molecular pathways that are crucial for proliferation and cell death [63]. It is confirmed that purine derivatives, belonging to the group of small compounds, could be potentially utilized to enhance the metabolic vulnerability of cancer cells, and these compounds have been shown to affect mitochondrial impairment [12,64] and induce oxidative stress [12]. *N*^6^-Furfuryladenosine kinetin riboside (KR) is a naturally occurring analogue of adenosine and has been reported to have a strong anticancer effect through the induction of apoptosis [12,64,65,66]. KR and several other purine derivatives have a unique mechanism of action that negatively affects the metabolic balance of the cells. RK can also induce energy imbalance through phosphorylation by ADK, the first enzyme of the salvage pathway of purine metabolism, which facilitates the toxicity of KR and leads to the accumulation of mono-, di-, and triphosphates [67]. Some of the purine analogues such 8-chloroadenosine and 2-chlorodeoxyadenosine have already presented hopeful results in clinical trials [68,69]. Similarly, KR has been used as one of the novel treatment alternatives for chronic lymphocytic leukemia (CLL), which include a new generation of purine derivatives [70], and it is also proposed as a potent inhibitor of the epithelial-to-mesenchymal transition (EMT) in human prostate cells [71]. Moreover, in reference to the current events related to the COVID-19 pandemic, KR is considered a promising candidate as an ACE2 receptor (angiotensin-converting enzyme 2) agonist [72].

In our previous study, we extended our observations of the anticancer activity of KR, and we showed that HepG2 cells undergo vast apoptosis, which is an immediate consequence of mitochondrial impairment, depletion of ATP, and disruption of oxidative parameters [12]. Following this path, in the present study, we investigated whether KR influences the generation of ROS in T98G cells and whether it could be used as a therapeutic option for treating GBM (Figure 1A). To develop our findings, we designed and synthesized new derivatives (Figure 2) of KR and examined whether these analogues were active in inducing OS in T98G cells in a similar concentration range to the corresponding KR. Several adenosine analogues are known to be good substrates of ADK, and the next step was to test, by molecular docking, how the addition of the furfuryl group at N^6^ would affect the affinity of those compounds to ADK and whether they could be used as KR derivatives with improved anticancer properties. Two of these compounds, namely, 8-azakinetin riboside (8-azaKR) and 7-deazakinetin riboside (7-deazaKR), were predicted to dock particularly well in the binding cavity of the ADK model (Figure 2B). Interestingly, 8-azaadenosine and some adenosine analogues containing a 7-deaza ring (sangivamycin and toyocamycin) are known to be phosphorylated by ADK and further incorporated into DNA and RNA, which contributes to their anticancer properties [37,38,39]. This characteristic makes the two adenine ring modifications particularly attractive to test in the context of KR derivatives. Furthermore, it has been shown that 8-azaadenosine is rapidly deaminated inside the cells and that the cytotoxicity of 8-azaadenosine is enhanced when cells are pretreated with an adenosine deaminase inhibitor [37]. The presence of a furfuryl group at N^6^ of 8-azaKR would most likely prevent the deamination reaction, which could contribute to the increased potency of 8-azaKR inside the cells as compared to 8-azaadenosine. The estimated binding energy indicated that the two KR derivatives might have similar affinity to ADK, like KR alone.

Cells are mostly cultured in monolayers (2D), and this cell environment may have an effect on the cellular response to external agents, e.g., drugs, and affect their cytotoxicity results [73]. Thus, we also performed analyses using the 3D cell cultures (spheroids), which mimic the natural microenvironment of growing tumor (TME), provide spatial cell–cell and cell–ECM (extracellular matrix) interactions [74], and also access the significantly stronger effects of therapeutics as compared to the response in 2D cultures [75]. The use of the T98G spheroids allowed us to select one derivative with a similar antitumor activity to KR (Figure 3, Figure 4 and Figure 5) and showed the comprehensive effect of the compounds. Before induction of the treatment, cell spheroids were fed for 3 days with a fresh medium and a medium additionally supplemented with compounds in order to preserve nutrient supply to the external cellular layers while the hypoxia/necrosis niche developed in the inner layer of increasing spheroids [76].

First, we performed confocal microscopy analysis to compare live and dead T98G cells forming spheroids after treatment with ribosides. The analysis revealed that all three ribosides affected cell viability in a dose- and time-dependent manner, which was observed as an increase in red fluorescence from dead cells (Figure 3). We also analyzed the effect of KR and its derivatives on the induction of intracellular and mitochondrial OS in 3D cultured T98G cells (Figure 4 and Figure 5). Spheroids as a research model create a microenvironment that supports tumor growth and enables closer interactions between cells. As mentioned above, spheroids show different responses to drugs, which might be due to cell–ECM interactions, which generate an ROS niche and constantly developing hypoxia. Consequently, the deprivation of oxygen within the tumor mass leads to higher ROS production, which creates an interconnected system [77,78].

OS enhances the invasiveness of the cells, but the treatment with the compounds increases the redox modifications within the GBM spheroids, as revealed by the inhibition of the proliferation and differentiation, leading to the rupture of the outer layer of the cell spheroids. A hypothesis describing the effect of ROS in cancer cells suggests that, when these cells are subjected to external ROS-producing agents, the intracellular ROS levels increase more easily to reach a threshold and induce cell death [35]. Taken together, these findings indicate that 7-deazaKR demonstrates greater anticancer activity on cell spheroids than 8-azaKR. Moreover, the effect of 7-deazaKR on induction of cell death and oxidative imbalance is comparable, but still not as significant as that of KR.

Next, the use of the monolayer cultures helped us primarily to comprehend the probable mechanism of action of KR and to choose 7-deazaKR to treat glioblastoma cells. We confirmed that these two adenosine analogues induce toxicity in T98G cells, inhibit cell proliferation, and initiate cell apoptosis in a dose-dependent manner (Figure 6D–G) through the salvage pathway of purine metabolism (Figure 6A). However, the effect on cellular proliferation was entirely averted when the cells were treated with 5-iodotubericidin, an inhibitor of ADK (Figure 6F,G); this indicated that the intracellular phosphorylation of the majority of purines is necessary to exert its cytotoxicity (Figure 6A,B) [63,79,80].

As mentioned above, the mechanism of purine derivatives in cancer cells is very complex and mainly depends on ADK [81] (Figure 6A). Following the entry into the cells, KR and 7-deazaKR are transformed into their monophosphate analogues, which affects the energy balance in cancer cells and is likely a straight consequence of the rapid and significant drop in the intracellular ATP level (Figure 6C) [12,65]. Currently, a similar response was reported for another well-known purine derivative, AICAR, which affects cellular metabolism, and its short-term effect is caused by the depletion of ATP in murine embryonic fibroblasts [82]. Moreover, the major processes involved in the activation of apoptosis are ATP depletion [55] and the induction of oxidative stress [83].

We have already proven that KR can trigger apoptosis through the induction of OS in HepG2 cells [12]. Furthermore, another naturally occurring compound—berberine—is increasingly emerging as a therapeutic agent for GBM treatment and exhibits a similar activity. This alkaloid is considered as a putative antitumor agent for targeting glioma, and, similar to KR and 7-deazaKR, it inhibits cell proliferation and, ultimately, induces cell death through OS [61,84,85].

Conducting therapies that affect the energy and redox balance in GBM cells is very challenging, with many limitations relying on the reduced susceptibility of cancer cells to medications [35]. It is established that nonmalignant cells maintain a moderate level of ROS, which contributes to the control of cell multiplication and differentiation. In cancer cells and even in GBM, increased intracellular ROS levels promote genome instability, hyperproliferation, and metastasis [86]. Therefore, we performed a comprehensive analysis of ROS generation in T98G cells after treatment with KR and 7-deazKR (Figure 7) and elucidated whether they might be promising alternatives for influencing the cellular redox environment in glioblastoma. We divided OS in two major groups: metabolic (Figure 7A–F) and genotoxic (Figure 8); moreover, because it is known that the antioxidant activity is essential for tumorigenesis [87,88], we evaluated the total content of GSH, which is a natural component of the antioxidant defense system (Figure 7G,H). ROS are generated permanently during cellular respiration and mediate the impulse of various signaling pathways [10]; however, the accumulation of ROS above the survival threshold leads to cell death [89]. In response to KR and 7-deazaKR, the byproducts of oxygen metabolism, i.e., intracellular ROS, are formed, and the redox balance of T98G cells was disturbed (Figure 7A,B). A greater effect was observed after KR treatment.

Similarly, in the study by Palma et al. [61], berberine treatment caused an increase in ROS levels and cell damage markers in U87MG cells. Moreover, it was shown that the flavonoid kaempferol can induce apoptosis of human glioma cells through the interference with cellular redox balance [90].

Mitochondria are one of the main sources of intracellular ROS, which produce ROS in the form of superoxide anions (O_2_^–^) as a byproduct of oxidative metabolism [86]. The mitochondrial ROS generation might lead to mtDNA damage and mutations, thereby causing respiratory chain dysfunction [91]. Thus, we performed an analysis of mitochondrial ROS generation and observed a significant increase in the superoxide level when KR concentration increased, whereas, after treatment with 7-deazaKR, the superoxide level slightly increased (Figure 7C,D). This finding confirmed our previous results that KR affects many cellular parameters, which indirectly results in mitochondrial stress and disruption of redox balance [12].

ROS are produced in several metabolic processes and interfere with different intracellular targets such as lipids, proteins, and DNA, resulting in genomic instability and finally cell rupture [35,92]. KR treatment induced a significantly high level of oxidized lipids, which was correlated with the lowering of the 590/510 ratio of the fluorescence intensity (Figure 7E,F). After 7-deazaKR treatment, we observed that the level of lipid peroxidation was comparable to that of the untreated T98G cells, with only a slight decrease in the ratio (Figure 7E).

Cellular lipid peroxidation is usually accompanied with ferroptosis, a newly discovered type of programmed cell death, which is activated when iron homeostasis in the cells is disrupted [93]. The induction of ferroptosis is mainly based on the Fenton reaction, wherein the accumulated iron (II) is oxidized into iron (III) by hydrogen peroxide, and lipid peroxides are in turn converted into ROS hydroxyl radical) [92]. Our results indicate that KR might be a potential inducer of ferroptosis and acts similar to erastin by inducing apoptotic cell death through ROS accumulation [94]. In contrast, Hu et al. [93] demonstrated that ferroptosis might play an important role in the resistance of gliomas to temozolomide, which might be confirmed on the basis of the results obtained after 7-deazaKR treatment. Ferroptosis is a very complex process with multiple factors, and it remains to be clarified whether it is involved in physiology and development of cancer cells or whether it occurs after pharmacological interventions (anticancer therapy) [93,95]. Thus far, few studies have reported that ferroptosis might involve the autophagy process, and it has also been described as a mechanism based on switching apoptosis to ferroptosis [96]. Thus, ferroptosis needs further detailed investigation in the context of purine analogues and its influence on lipid peroxidation.

It is known that ROS can be regulated by enzymatic scavengers, which are the key players of the antioxidant defense system that maintain intracellular redox balance through the reduction of ROS [97]. Among them, ROS homeostasis in vivo is mainly controlled by GSH that also participates in many other metabolic processes [98] such as cell differentiation, proliferation, immune response, and cell death [99]. GSH is present in three main forms: reduced GSH, which is the predominant form under physiological conditions, glutathione disulfide (GSSG), and glutathione–protein mixed disulfides (PSSG) [100]. GSH and other ROS scavengers exhibit a dual role in cancer cells, both maintaining a redox balance and promoting cancer progression and drug resistance in order to avoid cell death [99]. TMZ-resistant glioma cells have pronounced levels of glutathione reductase (GR) and GSH, and this is correlated with the regulation of redox status and drug response of GBM cells [35,88]. We also showed that, in response to KR and 7-deazaKR treatment, T98G cells show a rapid increase in total GSH content, which is likely a direct consequence of forcing the detoxification of elevated ROS (Figure 7G,H). In our previous study, we observed an opposite effect and significant depletion of reduced GSH in HepG2 cells after KR induction [12]. However, we proved that HepG2 cells show a greater adaptation to mitochondrial metabolism and that forcing oxidative phosphorylation increases their sensitivity to KR. We also confirmed that the metabolism of T98G cells mainly relies on anaerobic glycolysis, which may lead to drug resistance (Figure 1B,C). Thus, we have certainly shown that ROS and metabolism are strongly interlinked [101]. Our results demonstrate that ROS generation increased in T98G cells treated with KR and 7-deazaKR, possibly due to the activation of the antioxidant defense mechanisms and, thus, chemoresistance (Figure 7). We showed that treatment with an external compound disrupted the redox balance of T98G cells, and this might be linked to the increased induction of apoptosis (Figure 6D).

ROS accumulation could damage DNA directly and cause oxidative lesions; 8-oxo-dG could be a reliable biomarker of OS [42]. Under normal conditions, a genome has one 8-oxo-dG molecule per 10^5^–10^6^ guanosines, which is equivalent to thousands of 8-oxo-dG molecules per cell [102]. We found that all the tested compounds significantly elevated the number of 8-oxo-dG molecules as compared to control cells; this finding complements the above considerations and confirms that both the adenosine derivatives induce OS in T98G cells (Figure 8). Castro et al. [103] also demonstrated that a high dose of ascorbate caused genotoxic and metabolic OS in glioma cells.

We also examined the effect of KR and 7-deazaKR on the expression level of selected genes associated with OS induction and response in T98G cells. The expression levels were established by real-time PCR (Figure 9). We examined the genes specifically associated with the activation of the antioxidant defense system and determined the expression levels of genes related to OS and cellular welfare indicators. The changes in the expression level of the selected genes and the functions of the encoded proteins are summarized in Table 2. First, we confirmed our previous observations about enzymatic ROS scavengers and found that both KR and 7-deazaKR increase the expression level of these genes; this might be related to maintaining redox homeostasis and drug resistance in T98G cells [35,88]. We observed that, after treatment with the derivatives, the most significant effect was observed for SOD; a previous study also showed that the expression of *SOD2* and its related protein Sp1 increased in TMZ-resistant cells [104]. Upregulation of ROS scavengers is one of the signs of malignancy and resistance of GBM cells. Taken together, these findings indicate that the genes stimulating the production of antioxidant proteins are activated [105]. Thus, after treatment with KR and 7-deazaKR, we observed an increase in the expression level of genes related to OS response and confirmed that the NRF2 gene is a key regulator of detoxification and protects cells from DNA damage induction [42]; moreover, sestrins also act as stress-sensor proteins [106]. Furthermore, our results indicate that the upregulation of NF-KB is not only crucial for GBM cells to stimulate cell proliferation [107] but also as a part of the antioxidant defense system.

KR and 7-deazaKR also influenced the activation of the cellular welfare indicators such as SIRT2, PARP1, TNFA, and p53; however, both compounds decreased the expression level of PGC-1a, which is a crucial regulator of mitochondrial energy metabolism [108] and a modulator of mitochondrial biogenesis [109]. Cho et al., also indicated that PGC-1a is correlated with mitochondrial dysfunction in GBM, which may lead to tumor progression; thus, its decreased expression due to treatment with KR and 7-deazaKR is a desirable effect.

We have shown an indirect effect of KR and 7-deazaKR on the redox state of T98G cells, and this might be the beginning for further and more detailed studies on the use of these compounds in anticancer therapies. It has already been shown that KR can be a potential drug in personalized therapy of CLL [70], while in vivo experiments on mice have also been performed, indicating its anticancer properties, especially for human malignancies with dysregulation of cyclin D1 or D2 [79,110].

## 5. Conclusions

The use of the complementary cell-based assays allowed for the investigation of the effect of KR and its derivatives, 8-azaKR and 7-deazaKR, on the functions of T98G cells. The application of T98G cell spheroids was the starting point for further research, which enabled screening and the selection of one derivative-7-deazaKR. By performing detailed analyses, we compared the antitumor and pro-oxidative activity of KR and 7-deazaKR.

The present study aided to comprehend the mechanism of T98G cell resistance to KR and 7-deazaKR treatment, which is mainly correlated with the high metabolic rate of GBM cells and elevated ROS production. Our results indicate that both of these compounds upregulate the expression of genes associated with protection against OS and affect many cellular parameters (inhibition of cell proliferation, ATP depletion, impairment of antioxidant defense system, and induction of apoptosis), resulting in oxidative imbalance in T98G cells. Thus, we proved that purine analogues could be potentially used to exploit the oxidative sensitivity of cancer cells.

Taken together, the results of this study demonstrate that KR and 7-deazaKR are anticancer agents and might serve as putative alternatives for oxidative therapy focused on the cellular redox environment of glioblastoma cells.

## Figures and Tables

**Table 1:** List of primers used for real-time PCR analysis.

Gene Name		Full Name	Forward Primer (5′–3′)	Reverse Primer (5′–3′)	UPL No.
Control genes	ACTB	Actin Beta	CCAACCGCGAGAAGATGA	CCAGAGGCGTACAGGGATAG	64
	TBP	TATA-Box-Binding Protein	CGGCTGTTTAACTTCGCTTC	CACACGCCAAGAAACAGTGA	3
	PGK1	Phosphoglycerate Kinase 1	ACGCTACTGCATTCCTGCTT	ACTGTTTTGTGGGGTTTTTGTT	13
	HPRT1	Hypoxanthine Phosphoribosyltransferase 1	TGACCTTGATTTATTTTGCATACC	CGAGCAAGACGTTCAGTCCT	73
Target genes	CAT	Catalase	TCATCAGGGATCCCATATTGTT	CCTTCAGATGTGTCTGAGGATTT	76
	GSS	Glutathione Synthetase	CCTGCTAGTGGATGCTGTCA	TCATCCTGTTTGATGGTGCT	1
	SOD	Superoxide Dismutase	TCCATGTTCATGAGTTTGGAGAT	TCTGGATAGAGGATTAAAGTGAGGA	40
	SESN1	Sestrin 1	GGGCCGTTACCCCTACATTA	TTCACTAAGTAGGAGCACTGATGTC	46
	SESN2	Sestrin 2	TCCGCCACTCAGAGAAGG	GGAGGGCGTACAGCAGAG	68
	NRF2	Nuclear Factor Erythroid 2-Related Factor 2	CAGATGCCACAGTCAACACA	GGCTCAGCTATGAAAGCAGAA	9
	NFKB	Nuclear Factor Kappa B	ACCCAAGGACATGGTGGTC	AGCCCCTTATACACGCCTCT	47
	SIRT2	Sirtuin 2	TTCAAGCCAACCATCTGTCA	GCTCCAGGGTATCTATGTTCT	40
	PGC1a	PPARG Coactivator 1 Alpha	AAACGATGACCCTCCTCACA	TTCTTTTTGGAGGTGCATTTG	84
	PARP1	Poly(ADP-Ribose) Polymerase 1	GACAGGAAAGACAACAGACAAATC	GGGGTGATGTGTTTGAACTTG	7
	TNFA	Tumor Necrosis Factor-Alpha	CAGCCTCTTCTCCTTCCTGAT	GCCAGAGGGCTGATTAGAGA	40
	p53	Tumor Protein P53	TAGTGTGGTGGTGCCCTATG	CACATGTAGTTGTAGTGGATGGT	21

**Table 2:** Summary of changes in the expression level of genes analyzed by real-time PCR, including the function/action of the encoded protein, as well as their implication/entanglement in the main cellular processes. Up arrow (up-expression); Down arrow (down-expression), number of arrows correlates with the strength of expression increase/decrease.

Cellular Significance	Gene	Full Gene Name	Changes in Expression Level		Function of the Encoded Protein
			KR	7-deazaKR	
Enzymatic scavengers involved in the antioxidant defense	SOD	Superoxide Dismutase	↑↑↑	↑↑	Involved in the antioxidant defense against oxidative stress; SOD catalyzes the dismutation of superoxide anion to hydrogen peroxide.
	CAT	Catalase	↑↑	↑	The key antioxidant enzyme catalyzing the decomposition of hydrogen peroxide into water and oxygen.
	GSS	Glutathione Synthetase	↑	↑↑	The important enzyme of cellular antioxidant defense; involved in the second step of biosynthesis of glutathione (GSH), one of the functions of which is to protect cells from oxidative damage by free radicals.
Regulating factors related to oxidative stress	SESN1	Sestrin 1	↑	↑↑↑	Sestrins are induced by the p53 protein and play a role in the cellular response to DNA damage and oxidative stress.
	SESN2	Sestrin 2	no change	↑↑↑	
	NRF2	Nuclear Factor Erythroid 2-Related Factor 2	↑	↑↑↑	A transcription factor that controls the expression of antioxidant proteins involved in oxidative damage protection.
	NFKB	Nuclear Factor Kappa B	↑↑	↑↑↑	A transcription regulator that is activated by various intra- and extracellular stimuli, including oxidant free radicals.
Cellular welfare indicators	PGC-1a	PPARG Coactivator 1 Alpha	↓↓↓	↓↓↓	A transcriptional coactivator of the genes involved in energy metabolism. It interacts with and regulates the activities of nuclear respiratory factors (NRFs).
	SIRT2	Sirtuin 2	↑↑	↑↑	Involved in protection against various types of cellular stress related to oxidative stress (e.g., upregulates the expression of FOXO3 target gene, decreasing ROS level); involved in DNA repair.
	PARP1	Poly(ADP-Ribose) Polymerase 1	↑	↑↑	Involved in the regulation of the molecular events related to the recovery of cells from DNA damage.
	TNFA	Tumor Necrosis Factor-Alpha	↑↑↑	↑	Multifunctional proinflammatory cytokine, involved in the regulation of a wide spectrum of biological processes, including cell proliferation, differentiation, apoptosis, and lipid metabolism.
	p53	Tumor Protein P53	↑↑	↑↑↑	The encoded protein responds to diverse cellular stresses to regulate the expression of target genes, thereby inducing cell-cycle arrest, apoptosis, senescence, DNA repair, or changes in metabolism.

## Data Availability

Data can be provided upon request.
